# Significant overlap between human genome-wide association-study nominated breast cancer risk alleles and rat mammary cancer susceptibility loci

**DOI:** 10.1186/bcr3607

**Published:** 2014-01-27

**Authors:** Jennifer Sanders, David J Samuelson

**Affiliations:** 1 Department of Biochemistry and Molecular Biology, University of Louisville School of Medicine, Louisville, KY 40292, USA; 2 James Graham Brown Cancer Center, University of Louisville School of Medicine, Louisville, KY 40292, USA; 3 Center for Genetics and Molecular Medicine, University of Louisville School of Medicine, Louisville, KY 40292, USA; 4 Health Sciences Center, University of Louisville School of Medicine, Louisville, KY 40292, USA

## Abstract

**Introduction:**

Human population-based genome-wide association (GWA) studies identify low penetrance breast cancer risk alleles; however, GWA studies alone do not definitively determine causative genes or mechanisms. Stringent genome- wide statistical significance level requirements, set to avoid false-positive associations, yield many false-negative associations. Laboratory rats (*Rattus norvegicus*) are useful to study many aspects of breast cancer, including genetic susceptibility. Several rat mammary cancer associated loci have been identified using genetic linkage and congenic strain based-approaches. Here, we sought to determine the amount of overlap between GWA study nominated human breast and rat mammary cancer susceptibility loci.

**Methods:**

We queried published GWA studies to identify two groups of SNPs, one that reached genome-wide significance and one comprised of SNPs failing a validation step and not reaching genome- wide significance. Human genome locations of these SNPs were compared to known rat mammary carcinoma susceptibility loci to determine if risk alleles existed in both species. Rat genome regions not known to associate with mammary cancer risk were randomly selected as control regions.

**Results:**

Significantly more human breast cancer risk GWA study nominated SNPs mapped at orthologs of rat mammary cancer loci than to regions not known to contain rat mammary cancer loci. The rat genome was useful to predict associations that had met human genome-wide significance criteria and weaker associations that had not.

**Conclusions:**

Integration of human and rat comparative genomics may be useful to parse out false-negative associations in GWA studies of breast cancer risk.

## Introduction

Breast cancer is a complex disease characterized by environmental, genetic, and epigenetic factors. Due to the complexity of developing this disease a woman’s individual risk may vary greatly from population risk estimates. The familial relative risk of developing breast cancer increases with the number of affected relatives, suggesting that there is a strong genetic component associated with this disease [1,2]. High-penetrance breast cancer risk mutations such as those of *BRCA1* and *BRCA2* have been identified [3,4]. Population frequencies of mutations with high-penetrance toward risk are rare due to their severe effects on individuals and, thus, these mutations account for only a small percentage of population risk. Risk alleles with moderate penetrance and minor allele population frequencies of 0.005 to 0.01 (for example, *PALB2*) are estimated to account for approximately 3% of risk. Therefore, a majority of population-based breast cancer risk is likely explained by low penetrance alleles with rare to common population frequencies [5].

Genome-wide association (GWA) studies have been used to identify several low penetrance breast cancer risk alleles [6]. Due to a need to control for numerous multiple comparisons made in GWA studies, a Bonferroni correction based *P*-value cut-off of ≤1 × 10^-7^ is typically required for an association to be considered genome-wide significant. It has been suggested that this approach is too stringent as it may result in many false negative associations [7]. Furthermore, while GWA studies are unbiased approaches to identify genomic regions associated with breast cancer risk, these epidemiology-based approaches cannot easily determine risk genes or genetically determined mechanisms of susceptibility. Currently, only a small percentage of breast cancer heritability is explained by published studies suggesting that considerable genetic variation associated with breast cancer risk remains to be identified [5,8].

Comparative genetics between rats and humans has also been used to identify breast cancer risk alleles [9]. In general, the laboratory rat is a good experimental organism to model breast cancer. Compared to induced mammary tumors in mice, rats develop mammary carcinomas of ductal origin, which is similar to a majority of human breast cancers. Also, rat mammary tumors are responsive to estrogen, just as are a majority of human breast tumors [10,11]. Most importantly, the laboratory rat is a versatile organism to study breast cancer susceptibility, as experiments can be controlled at genetic and environmental levels. Inbred rat strains exhibit differential susceptibility to chemically induced carcinogenesis using 7,12-dimethylbenz[a]anthracene (DMBA) [10,12-14]. Copenhagen (COP) and Wistar- Kyoto (WKY) rat strains are resistant to DMBA, *N*-Nitroso-*N*-methylurea (NMU), and oncogene induced mammary carcinomas, while the Wistar- Furth (WF) rat strain is susceptible.

Previous genetic studies using rats have identified eight *Mammary carcinoma susceptibility* (*Mcs*) loci, named *Mcs 1–8*[15-18]. A (WFxCOP)_F1_ x WF backcross design was used to identify *Mcs 1–4*. Copenhagen alleles at *Mcs 1–3* are associated with decreased mammary tumor multiplicity, while the *Mcs 4* COP allele is associated with increased tumor development [15]. Further analysis of the *Mcs1* locus using WF.COP congenic lines, spanning different regions of the quantitative trait locus (QTL), identified three independent loci associated with mammary carcinoma susceptibility, named *Mcs 1a-c*[17]. Another linkage analysis study using WF and WKY rat strains revealed four additional QTLs associated with mammary carcinoma susceptibility, named *Mcs 5–8*. Additionally, a modifier of *Mcs8, Mcsm1*, partially counteracts the resistance phenotype conferred by *Mcs8*[16,18]. Further analysis of the *Mcs5* locus using WF.WKY congenic rat lines resulted in the identification of four subloci named *Mcs5a1*, *Mcs5a2*, *Mcs5b* and *Mcs5c*[9,19]. Additional linkage analysis using the SPRD-Cu3 rat strain (DMBA-induced mammary carcinogenesis susceptible) and the resistant WKY rat strain resulted in the identification of three more rat QTLs associated with mammary cancer named *Mcstm1*, *Mcstm2/Mcsta2* and *Mcsta1*[20,21]. Several rat genomic regions that associate with mammary cancer susceptibility were identified using beta-estradiol instead of DMBA to induce carcinogenesis. These QTLs were identified using the August Copenhagen Irish (ACI) rat strain, which is susceptible to beta-estradiol carcinogenesis and the COP and Brown Norway (BN) rat strains, which are resistant. These loci are named *Estrogen-induced mammary cancer* loci or *Emca 1*–*2* and *Emca 4–8*[22,23].

Comparative genomics between human breast and rat mammary cancer risk alleles will continue to be warranted, especially if appreciable overlap in genetic susceptibility exists between these species. In this study, genomic locations of human breast cancer risk GWA study-identified polymorphisms were compared to the rat genome to determine if positive associations were more often located at orthologs to rat mammary cancer risk loci than at randomly selected regions not known to be associated with rat mammary cancer susceptibility.

## Methods

### Converting rat mammary cancer associated loci to human orthologous regions

No research animals were used in this work. Previously published information on rat mammary cancer associated loci was used. Human orthologous regions of rat regions that associate with mammary cancer susceptibility listed in Table 1 were determined using the ‘In other genomes (convert)’ function available at the University of California Santa Cruz (UCSC) genome browser [24]. Rat Nov. 2004 (Baylor 3.4/rn4) and human Feb. 2009 (GRCh37/hg19) genome assemblies were used. If a rat mammary cancer locus split into multiple human orthologous regions, we noted all orthologous regions until they reached less than 1% of the bases and spanned less than 1% of the original rat mammary cancer locus using the UCSC genome browser.

**Table 1:** Location of rat mammary cancer susceptibility loci and human orthologous regions used in this study

**Rat *Mcs *** **locus (Overlap)**	**Boundary markers**	**Rat chr**	**Region(UCSC rat assembly 2004)**	**Reference**	**Human orthologous region (UCSC human assembly 2009)**
**DMBA induced mammary carcinogenesis**					
***Mcs1a***	D2Mit29 to D2Uwm14	RNO2	5,601,528- 10,539,344	Haag *et al*. [17]	*Chr5*: 89,216,702-93,113,337
***Mcs1b***	ENSRNOSNP2740854 to g2Ul2-27	RNO2	42,364,155-44,195,382	DenDekker *et al*. [25]	*Chr5*: 54,816,178-57,003,049
***Mcs1c***	D2M13Mit286 to D2Uia5	RNO2	13,909,383- 20,666,092	Haag *et al*. [17]	*Chr5*: 81,891,633-86,857,442
					*Chr5*: 86,171,198-86,251,067
***Mcs2*** (overlaps *Mcs6, Emca4*)	D7rat39 to D7Uwm12	RNO7	4,936,704-86,028,057	Sanders *et al*. [18]	*Ch12*: 57,316,160-108,177,690
					*Chr8*: 97,242,984-115,650,989
					*Chr19*: 281,161-2,497,331
					*Chr19*: 15,059,910-15,808,112
***Mcs3***	D1Rat27 to D1Mit12	RNO1	90,282,174-156,954,117	Shepel *et al*. [15]	*Chr15*: 80,282,370-102,265,870
					*Chr15*: 25,574,935-28,567,541
					*Chr11*: 17,403,456-22,898,646
					*Chr11*: 74,958,193-89,350,902
					*Chr19*: 48,799,986-51,921,957
					*Chr19*: 28,701,413-30,656,003
***Mcs4***	D8Rat164 to D8Rat108	RNO8	28,414,100-72,403,639	Shepel *et al*. [15]	*Chr11*: 107,453,990-132,383,506
					*Chr15*: 62,105,069-76,028,735
					*Chr15*: 76,091,658-78,185,872
					*Chr15*: 78,380,119-78,998,961
					*Chr15*:51,349,646-51,942,505
***Mcs5a1***(overlaps *Mcstm1, Emca8*)	SNP-61634906 to SNP- 61666918	RNO5	61,634,727-61,666,739	Samuelson *et al*. [9]	*Chr9*: 37,562,516-37,589,491
***Mcs5 a2***(overlaps *Mcstm1, Emca8*)	SNP-61667232 to gUwm23-29	RNO5	61,667,053-61,751,614	Samuelson *et al*. [9]	*Chr9*: 37,590,988-37,654,512
***Mcs5b***(overlaps *Mcstm1, Emca8*)	gUwm50-20 to D5Got9	RNO5	65,498,190-67,464,050	Samuelson *et al*. [19]	*Chr9*: 103,492,712-105,220,552
***Mcs5c***(overlaps *Mcstm1, Emca8*)	gUwm74-1 to gUwm54-8	RNO5	81,118,457-81,295,367	Veillet *et al*. [26]	*Chr9*: 118,231,525-118,416,951
					*Chr12*: 72,033,141-72,033,263
***Mcs6***(overlaps *Mcs2*)	D7Rat171 to gUwm64-3	RNO7	22,382,725-55,384,873	Sanders *et al*. [18]	*Chr12*: 71,270,266-105,502,699
***Mcs7***(overlaps *Mcsta1*)	D10Got124 to gUwm58-136	RNO10	89,575,060-100,335,500	Cotroneo *et al*. [27]	*Chr17*: 40,183,547-67,946,104
***Mcs8***	D14Mit1 to D14Rat99	RNO14	12,386,493-26,416,791	Lan *et al*. [16]	*Chr4*: 65,556,457-81,559,483
***Mcsm1***(overlaps *Emca7*)	D6Mit9 to D6Rat12	RNO6	34,039,303-114,032,192	Lan *et al*. [16]	*Chr14*: 25,151,530-80,417,386
					*Chr2*: 334,41-18,603,019
					*Chr7*: 12,561,599-19,619,365
					*Chr7*: 107,770,320-111,916,436
***Mcstm1***(overlaps *Mcs5,Emca1, Emca8)*	D5rat124 to *Pla2g2a*	RNO5	19,206,257-157,657,360	Piessevaux *et al*. [21]	*Chr1:* 20,301,931-59,012,763
					*Chr1:* 59,119,520-67,602,141
					*Chr9:* 27,325,071-123,488,955
					*Chr6:* 87,792,854-100,245,025
					*Chr8:* 87,055,841-97,247,307
					*Chr8:* 58,994,818-62,700,945
***Mcstm2***(overlaps *Emca2*)	D18Wox8 to D18Rat44	RNO18	32,458,819-86,863,412	Piessevaux *et al*. [21]	*Chr18:* 10,202,644-13,129,349
					*Chr18:* 41,356,963-54,158,113
					*Chr18:* 54,267,924-58,201,561
					*Chr18:* 66,912,039-78,010,606
					*Chr5:* 112,300,500-130,363,372
					*Chr5:* 142,780,151-147,624,793
					*Chr5:*147,647,196-150,176,352
***Mcsta1***(overlaps *Mcs7*)	D10Rat91 to *D10Rat97*	RNO10	9,762,188-108,776,963	Piessevaux *et al*. [21]	*Chr5:* 130,482,861-173,663,969
					*Chr5:* 177,530,539-180,675,650
					*Chr17:* 690,639-15,624,409
					*Chr17:* 16,916,926-20,222,700
					*Chr17:* 25,525,650-78,247,249
					*Chr16:* 78,402-6,094,950
***β*-estradiol induced mammary carcinogenesis**					
***Emca1***(overlaps *Mcstm1, Emca8*)	D5Rat53 to D5Rat57	RNO5	103,677,474-155,121,024	Gould *et al*. [22]	*Chr1:* 23,607,020-59,012,763
					*Chr1:* 59,119,520-67,602,141
					*Chr9:* 17,037,252-27,300,264
***Emca2***(overlaps *Mcstm2*)	D18Rat27 to D18Rat43	RNO18	18,562,643-66,652,947	Gould *et al*. [22]	*Chr5:* 110,259,180-130,363,372
					*Chr5:* 137,224,929-147,624,793
					*Chr5:* 147,647,196-150,176,352
					*Chr18:* 10,202,644-13,129,349
					*Chr18:* 35,982,130-41,016,602
					*Chr18:* 52,597,120-54,158,113
					*Chr18:* 54,267,924-58,201,561
					*Chr2:* 127,805,417-128,786,719
***Emca4***(overlaps *Mcs2*)	D7Rat44 to D7Rat15	RNO7	66,201,980-107,428,439	Schaffer *et al*. [23]	*Chr8:* 97,242,984-137,409,536
					*Chr12:* 57,316,160-59,093,375
***Emca5***	D3Rat227 to D3Rat210	RNO3	41,054,012-171,063,335	Schaffer *et al*. [23]	*Chr20:* 1,746,912-62,907,504
					*Chr2:* 110,841,402-113,650,057
					*Chr2:* 159,530,076-188,395,371
					*Chr11:* 26,296,319-57,753,858
					*Chr15:* 32,905,485-34,664,466
					*Chr15:* 34,933,152-51,298,144
***Emca6***	D4Rat14 to D4Rat202	RNO4	41,729,583-159,115,617	Schaffer *et al*. [23]	*Chr7:* 23,252,368-33,103,107
					*Chr7:* 115,026,301-150,558,396
					*Chr3:* 88,756-12,883,445
					*Chr3:* 13,004,609-15,163,132
					*Chr3:* 64,018,604-75,322,612
					*Chr3:* 125,977,400-128,219,297
					*Chr2:* 68,713,643-89,165,869
					*Chr4:* 89,504,626-95,273,083
					*Chr4:* 120,978,632-122,320,931
					*Chr12:* 156,786-2,821,588
					*Chr10:* 43,277,230-46,218,580
***Emca7***(overlaps *Mcsm1*)	D6Rat68 to D6Rat81	RNO6	2,802,670-111,967,837	Schaffer *et al*. [23]	*Chr14:* 25,151,530-78,362,253
					*Chr2:* 33,441-35,642,893
					*Chr2:* 38,644,737-51,698,454
					*Chr7:* 12,561,599-19,619,365
					*Chr7:* 105,197,211-111,916,436
***Emca8***(overlaps *Mcs5*, *Mcstm1, Emca1*)	D5Rat134 to D5Rat37	RNO5	52,434,178-148,460,381	Schaffer *et al*. [23]	*Chr9:* 6,756,013-27,300,264
					*Chr9:* 27,925,947-123,488,955
					*Chr1:* 33,159,021-59,012,763
					*Chr1:* 59,119,520-67,602,141

### Random rat regions

To determine if human GWA study-identified polymorphisms map to rat mammary cancer loci more frequently than to random regions of the rat genome, we selected rat genome segments that have not shown an association with mammary cancer risk. These rat genomic regions were named ‘random rat regions’ and are listed in Table 2. We initially focused on fourteen *Mcs/Mcsm* regions with an average size of 22,322,710 bps as these are generally smaller in size than other rat mammary cancer associated loci identified. Fourteen random rat genome regions, each 22,322,710 bps in size were used for comparison. Random rat regions were selected by picking a chromosome using a random number generator function of Microsoft Excel. The range of chromosomes entered into the random number generator function was 1 through 21 (rats have 21 chromosomes, including a sex chromosome). The start position for each random rat region was determined using a random number generator function of Excel. The rat genome is 2.75 Gb in size [28] or 130,952,381 bp per chromosome if divided equally across chromosomes. Therefore, values for the rat genome start-position were chosen from 1 to 130,952,381 using a random number generator. Following, 22,322,709 bps were added to each random start position to obtain the desired full length. The 14 random rat genome regions were then entered into the UCSC genome browser, and the human orthologous regions were determined using the ‘in other genomes (convert)’ function, as described above [24]. Randomly-generated rat genome segments were used as controls if the human orthologous segment did not contain a sequence that was also orthologous to a known rat mammary cancer associated locus. We also verified, using the UCSC genome browser, that human orthologous regions to random rat regions were not located at human centromeric regions, as genetic variation in these chromosomal regions is underrepresented in GWA studies [29,30]. Total sizes and percentages of rat genome covered by rat mammary cancer loci and random genome regions used are in Table 3.

**Table 2:** Random rat genomic segments and human orthologous regions used in this study

**Rat *Mcs *** **locus**	**Rat *chr***	**Region (UCSC rat assembly 2004)**	**Human orthologous region (UCSC human assembly 2009)**
**Random rat region 1**	RNO9	20,000,000-44,322,711	*Chr2:* 97,158,323-106,711,249
			*Chr6:* 56,223,874-73,919,999
			*Chr2:* 189,182,486-189,878,065
			*Chr13:* 103,235,577-103,556,495
			*Chr2:* 128,848,569-129,254,860
**Random rat region 2**	RNO15	60,000,001-84322711	*Chr13:* 53,226,266-74,878,291
			*Chr13:* 42,064,282-42,529,444
**Random rat region 3**	RNO16	68,621,246-92,943,956	*Chr13:* 103,539,456-115,092,822
			*Chr8*: 36,627,241-42,308,840
			*Chr8:* 638,582-6,693,649
			*Chr8:* 42,690,588-43,056,179
			*Chr13:* 52,753,969-53,050,606
**Random rat region 4**	RNO9	91,398,460-115,721,170	*Chr5:* 98,385,946-110,062,886
			*Chr18:* 612,848-9,957,727
			*Chr2*: 240,340,012-242,806,427
**Random rat region 5**	RNO13	55,373,307-79,696,017	*Chr1:* 169,844,936-194,938,667
**Random rat region 6**	RNO11	39,408,000-63,730,710	*Chr3:* 95,108,010-118,895,417
**Random rat region 7**	RNO17	68,384,015-92,706,72	*Chr10:* 138,740-22,530,353
			*Chr1:* 236,673,870-240,084,642
**Random rat region 8**	RNO3	12,585,543-36,908,253	*Chr2:* 140,246,548-155,465,845
			*Chr9:* 123,526,091-129,443,210
**Random rat region 9**	RNO19	34,130,390-58,453,100	*Chr16:* 66,968,878-90,107,058
			*Chr10:* 33,502,588-35,153,585
			*Chr1:* 229,402,942-235,324,796
			*Chr4:* 150,548,912-150,855,848
**Random rat region 10**	RNO12	18,203,110-42,525,820	*Chr12:* 110,503,298-120,870,994
			*Chr12:* 121,578,435-132,335,900
			*Chr7:* 66,878,689-71,941,664
			*Chr7:* 101,137,811-102,184,451
			*Chr7:* 99,995,220-100,350,712
			*Chr7:* 72,707,443-74,223,683
			*Chr7:* 75,027,443-76,145,496
**Random rat region 11**	RNO20	30,416,373-54,739,083	*Chr6:* 101,086,446- 116,620,662
			*Chr6:* 117,266,139-123,147,126
			*Chr2:* 109,065,537-109,613,060
			*Chr6:* 116,688,407-116,905,609
**Random rat region 12**	RNO13	955,085-25,277,795	*Chr18:* 58,351,906-63,553,937
			*Chr2:* 124,758,685-125,682,595
**Random rat region 13**	RNO1	1,136,860- 25,459,569	*Chr6:* 128,011,342-150,185,813
			*Chr6*: 123,315,387-124,317,854
**Random rat region 14**	RNO2	182,078,762-206,401,472	*Chr1:* 107,259,608-154,441,176

**Table 3:** Total size and percentage of rat genome covered by rat mammary cancer loci and random rat regions

**Region**	**Loci**	**Total size (bases)**	**Total overlapping bases**	**Total unique bases**	**Rat genome portion (based on total unique bases)**
***Mcs/Mcsm *** **only**	14	345,323,605	33,002,148	312,321,457	11.4%
**All known ratmammary cancer loci**	24	1,230,487,116	325,386,323	905,100,793	32.9%
**Random rat regions**	14	312,517,940	-	312,517,940	11.4%

### Determining human GWA study nominated polymorphisms

Human breast cancer risk GWA studies considered were published through March 2013. In the first round of analysis we picked GWA studies with a clearly defined study population of subjects of European descent. In the second round of analysis, the defined population was broader and included studies that tested populations of non-European descent. Studies that included non-European descent populations were subdivided into respective populations used. The GWA studies evaluated are listed in Tables 4 and 5. Results from GWA studies used consisted of multiple stages (two to four stages) to evaluate breast cancer risk association. In our analysis, all SNPs that entered the final stage of their respective study were compared in the rat genome. A tested SNP was called either ‘associated’ if it reached genome wide significance in its respective study or ‘potentially associated’ if it failed to meet the respective study statistical criterion following the final stage of analysis. Conventionally, a *P*-value level for an association to be considered statistically significant in a GWA study is 1 × 10^-7^. This stringency is to protect from false-positives due to multiple comparisons on a genome-wide scale. It has been argued that this low *P*-value requirement results in numerous false negative associations [7]. Therefore, we queried supplemental material of each published GWA study considered and picked polymorphisms that failed the validation stage in the respective study. We also included polymorphisms that did reach genome-wide significance. We considered 533 SNPs from studies that included populations of European descent, and 285 SNPs from studies of non-European descent populations. All SNPs used in this analysis are listed in Additional file 1. Human genomic locations of polymorphisms were found using dbSNP (GRCh37 assembly) [31]. These were compared to locations of the human orthologous regions of rat mammary cancer loci and random rat regions. If a polymorphism mapped to a region of interest, the name, location, odds ratio, 95% confidence interval, and *P*-value were noted.

**Table 4:** Breast cancer risk genome-wide association studies using populations of European descent

**GWAS**	**Population**	**Stages**	**Cases/controls stage 1**	**Cases/controls stage 2**	**Cases/controls stage 3**	**Cases/controls stage 4**	**Study *P*-value cut-off for significance**
Ahmed *et al*. [32]	European descent	4	390/364	3,990/3,928	3,878/3,928	33,134/36,141	*P* < E-07
Antoniou *et al*. [33]	European descent	2	1,193/1,190	5,986/2,974			*P* < E-07
Easton *et al*. [34]	European descent	3	408/400	3,990/3,916	21,860/22,578		*P* < E-07
Fletcher *et al*. [35]	European descent	3	3,981/2,365	4,804/3,936	4,237/5,044		-
Garcia-Closas *et al*. [36]	European descent	2	4,193/35,194	6,514/41,455			*P* < 5E-08
Gaudet *et al*. [37]	European descent	2	899/804	1,264/1,222			*P* < E-05
Ghoussaini *et al*. [38]	European descent	2	56,989/58,098	69,564/68,150			*P* < E-04
Haiman *et al*. [39]	European descent/African descent	2	African descent (1,004/2,745), European descent (1,718/3,670)	European descent (2,292/16,901)			-
Hunter *et al*. [40]	European descent	2	1,145/1,142	1,776/2,072			*P* < 2E-05
Li *et al*. [41]	European descent	2	617/4,583	1,011/7,604			*P* < E-05
Li *et al*. [42]	European descent	2	2,702/ 5,726	?			*P* < E-06
Mavaddat *et al*. [43]	European descent	2	4,470/4,560	?			*P* < 5E-02/6.25E-03
Michailidou *et al*. [44]	European descent	2	10,052/12,575	45,290/41,880			*P* < 5E-08
Murabito *et al*. [45]	European descent	1	250/1,345				*P* < 5E-08
Sehrawat *et al*. [46]	European descent	2	348/348	1,153/1,215			*P* < 6.4E-08
Stacey *et al*. [47]	European descent	2	1,600/11,563	4,554/17,577			*P* < E-07/*P* < 6.8E-08
Stacey *et al*. [48]	European descent	2	6,145/33,016	5,028/32,090			-
Thomas *et al*. [49]	European descent	3	1,145/1,142	4,547/4,434	4,078/5,223		*P* < 5E-07
Turnbull *et al*. [50]	European descent	2	3,659/4,897	12,576/12,223			*P* < E-04

**Table 5:** Breast cancer risk genome-wide association studies of non-European descent populations

**GWAS**	**Population**	**Stages**	**Cases/controls stage 1**	**Cases/controls stage 2**	**Cases/controls stage 3**	**Cases/controls stage 4**	**Study *P*-value cut-off for significance**
Cai *et al*. [51]	Asian descent	4	2,062/2,066	4,146/1,823	6,436/6,716	4,509/6,338	-
Chen *et al*. [52]	African- American descent	2	3,153/2,831	3,607/11,330			*P* < 5E-08
Gold *et al*. [53]	Ashkenazi Jewish descent	3	249/299	950/979	243/187		*P* < E-05
Haiman *et al*. [39]	African descent/ European descent	2	African descent (1,004/2,745), European descent (1,718/3,670)	European descent (2,292/16,901)			-
Kim *et al*. [54]	Asian descent	3	2,273/2,052	2,052/2,169	1,997/1,676		*P* < 5E-04
Long *et al*. [55]	Asian descent/ European descent	3	2,073/2,084	4,425/1,915	Asian descent (6,173/6,340), European descent (2,797/2,662)		-
Long *et al*. [56]	Asian descent	4	2,918/2,324	3,972/3,852	5,203/5,138	7,489/9,934	*P* < 5E-08
Zheng *et al*. [57]	Asian descent	3	1,505/1,522	1,554,1,576	3,472/900		*P* < 5E-08
Zheng *et al*. [58]	Asian descent	2	23,637/25,579				*P* < 1.5E-03

### Statistics

The number of human polymorphisms that mapped to orthologous regions containing rat mammary cancer loci (observed) was compared to the number of human polymorphisms that mapped to random rat regions (expected) using a chi-square analysis with one degree of freedom. Several rat mammary cancer loci overlap extensively and subsequently several human polymorphisms mapped to multiple rat loci. Currently, it is not known if these overlapping rat mammary cancer loci would fine-map to the same locus or independent loci. For this study, human polymorphisms mapping to overlapping rat mammary cancer susceptibility associated sequences were counted only once. For analysis of associated (passed genome-wide significance level) versus potentially associated (did not pass genome-wide significance level) associations, a logistic regression was performed using SYSTAT 13 statistical software. A threshold of associated or potentially associated was used as the independent variable and outcome was either the SNP mapped to a rat mammary cancer locus or it mapped to a random rat region.

## Results

### Significantly more breast cancer risk GWA study nominated SNPs are located at orthologs of rat *Mcs/Mcsm* loci compared to random rat genomic regions

**Figure 1:**
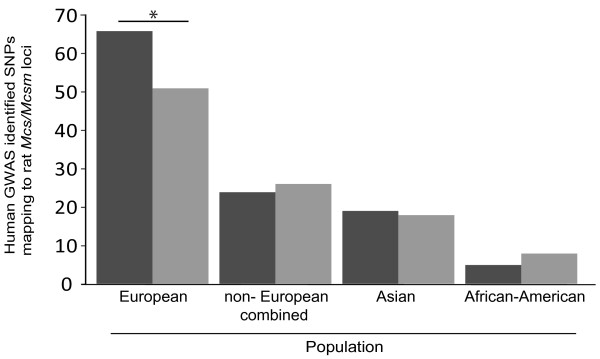
**Number of breast cancer risk GWA study nominated SNPs mapping to rat *Mcs/Mcsm *regions.** Number of GWA study nominated SNPs mapping to orthologs of rat *Mcs/Mcsm* loci and rat random regions. Dark grey columns represent the number of GWA study nominated human SNPs mapping to the human orthologous regions of the *Mcs/Mcsm* loci. Light grey columns represent the number of GWA study nominated human SNPs mapping to the human orthologous regions of the random rat control regions. The difference between risk associated SNPs mapping to rat *Mcs/Mcsm* and random rat regions was statistically significant for European populations. Asterisk indicates *P*-value <0.05 using chi-square analysis with number of SNPs mapping to *Mcs/Mcsm* set as the observed value and number of SNPs mapping to random rat regions as the expected value. GWA, genome-wide association.

We picked 28 GWA studies of breast cancer risk in which well-defined populations were analyzed (Table 4). Physical locations of polymorphisms that failed the final validation step and polymorphisms that reached genome-wide significance were determined using dbSNP [31]. We included SNPs that failed the final validation step of the respective study, because it has been suggested that many true associations are ruled out in a GWA study due to stringent statistical analysis methods [7]. We determined if sequences containing these polymorphisms were located at either a human genome region orthologous to a known rat mammary cancer locus or to a randomly selected region of the rat genome. Our goal was to determine if GWA study-nominated potentially-associated (did not pass final validation) and associated (genome-wide significant) risk polymorphisms, together map more often to human orthologous regions of rat mammary cancer susceptibility loci than to randomly selected rat genome segments of similar size. If yes, it would suggest that human GWA information combined with rat genetic susceptibility information is broadly useful to determine true genetic associations. Overall, rat *Mcs/Mcsm* loci are mapped to shorter genomic segments than other rat mammary cancer risk loci; therefore, we first compared overlap between human GWA study nominated breast cancer risk SNPs and rat *Mcs/Mcsm* loci to overlap of human associated SNPs with randomly selected rat genomic regions not known to contain mammary cancer susceptibility loci (Figure 1). Human GWA studies were grouped by population of descent for comparison. There was a significant difference between the number of GWA study nominated SNPs mapping to rat *Mcs/Mcsm* loci compared to random rat regions in studies analyzing populations of European descent (66 SNPs to 51 SNPs respectively, *P*-value <0.05). Human GWA study identified polymorphisms located at orthologs of rat loci are listed in Additional file 2. This result supports previous studies indicating rat genetic susceptibility is useful to predict and study human breast cancer risk loci. There was no difference in Asian or African-American descent populations. This is likely due to a limited number of published population-based breast cancer risk genetic-association studies using these populations.

### Breast cancer risk GWA study nominated polymorphisms map more often to orthologs of all known rat mammary cancer loci than to randomly selected regions

**Figure 2:**
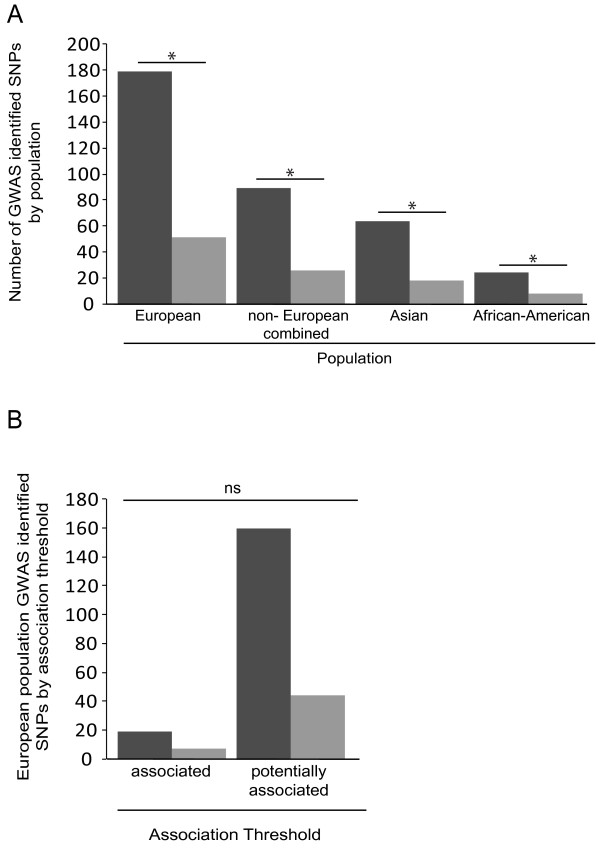
**Number of breast cancer risk GWA study nominated SNPs mapping to orthologs of rat mammary cancer loci or randomly selected rat genomic segments.** Dark grey columns indicate GWA study nominated SNPs that map to human orthologous regions of rat mammary cancer loci. Light grey columns indicate GWA study nominated SNPs that mapped to human orthologous regions of randomly selected rat genomic regions. **A)** Studies by population descent. Asterisks indicate statistical significance (*P* <0.01). The difference between risk associated SNPs mapping to rat mammary cancer loci and random rat regions in studies of European, Asian and African-American descent populations was significant (*P*-values <0.01 using chi-square analysis with the number of SNPs mapping to rat mammary cancer loci set as the observed value and the number of SNPs mapping to random rat regions as the expected value). **B)** Associated and potentially associated SNPs identified in populations of European descent that mapped to rat regions of interest were compared using logistic regression. Threshold of association was not a significant predictor of whether a SNP mapped to an ortholog of a rat mammary cancer locus or a random rat region. ‘ns’ indicates a comparison was not statistically significant. GWA, genome-wide association.

Next, we included additional rat mammary cancer susceptibility loci that have been identified, but span large genomic segments. Loci added were *Mcstm1, Mcstm2, Mcsta1, Emca1-2* and *Emca4-8*[20-23]. The same random rat genomic regions used previously were used in this analysis to be consistent. Respectively, 179 and 51 GWA study nominated polymorphisms were located in human orthologous regions to rat mammary cancer loci and randomly selected rat regions (Figure 2A) when studies using populations of European descent were considered. This difference was statistically significant (*P* <0.01). Note, some rat mammary cancer loci identified in independent studies have long regions of overlap. Consequently, several human GWA study identified polymorphisms mapped to human sequence orthologous to overlapping rat susceptibility loci. As it is not known if these rat loci contain unique sub-loci, human risk associated polymorphisms mapping to overlapping rat regions were counted only once. The size of the rat genome covered by all known rat mammary cancer susceptibility loci compared to control loci was disproportionate (Table 3). However, the ratio of breast cancer risk associated human SNPs at orthologs to rat mammary cancer susceptibility loci to SNPs at random segments was higher than the ratio of susceptibility loci bases to random bases (3.5 versus 2.9). This result was relatively proportionate to the previous result when only rat *Mcs/Mcsm* loci were considered (1.29 for *Mcs/Mcsm* and 1.21 for all susceptibility loci), suggesting that a potential bias was not introduced by the increase in total genomic coverage.

Not surprisingly, only 179 of 533 or 33.6% of the total human GWA study identified SNPs using populations of European descent were located at orthologs to rat mammary cancer associated loci. It is notable that 57 of the 533 total SNPs evaluated were reported in more than one GWA study; a majority of these were potential associations that failed the final validation step of the respective study. These results further suggest that there are several breast cancer risk associated SNPs not reaching genome-wide statistical significance in human population-based genetic studies.

Since more breast cancer risk polymorphisms nominated from GWA studies of populations of European descent mapped to orthologs of rat mammary cancer loci than to randomly selected regions of the rat genome, we determined if this was the case for association studies using non-European descent populations. We queried the nine GWA studies of populations of non-European ancestry that are listed in Table 5. These were GWA studies using populations of African, African-American, Ashkenazi Jewish, and Asian descent; however, only polymorphisms from studies using African-American, Ashkenazi Jewish and Asian descent populations mapped to any of the human orthologous segments to rat genomic regions picked for this study. First, results from all studies of non-European descent populations were combined (Figure 2A). Eighty-nine risk associated SNPs mapped to orthologs of rat mammary cancer loci and 26 SNPs were located at randomly selected rat regions. Next, studies using populations of Asian, Ashkenazi Jewish and African-American descent were considered separately. This resulted in 64 Asian descent population SNPs mapping to orthologs of rat mammary cancer loci and 18 SNPs to random rat regions. Twenty-four SNPs identified in studies of African-American descent populations were located at orthologs to rat mammary cancer loci and eight SNPs in random rat regions. The difference between rat mammary cancer loci and random regions was statistically significant (*P* <0.01) for both populations (Figure 2A). Interestingly, one SNP from a study of an Ashkenazi Jewish population mapped to the human orthologous region of rat *Mcsta1,* but no GWA study nominated SNP from that study mapped to a rat random region [53]. The lack of human SNPs mapping to orthologs of rat mammary cancer loci from populations of African and Ashkenazi Jewish decent may be due to a limited number of studies conducted on these populations. On the other hand, it may indicate that susceptibility alleles different from those currently identified in laboratory rats are segregating in these populations. Out of 285 SNPs considered from studies using populations of non-European descent, 89 SNPs or 31% mapped to orthologs of rat mammary cancer loci (Additional file 2). Fifteen risk-associated SNPs were represented in more than one human GWA study.

Next, GWA-study nominated variants from populations of European descent were separated by associated (reached genome-wide significance) and potentially associated (did not reach genome-wide significance after the final stage) variants (Figure 2B). Nineteen associated SNPs were located at rat mammary cancer loci compared to seven SNPs that mapped to random rat regions. Comparatively, 160 potentially associated SNPs mapped to rat mammary cancer susceptibility loci compared to 44 SNPs that mapped to random rat regions. A logistic regression was performed using threshold of association (associated or potentially associated) as the independent variable and rat genome location (ortholog of a rat mammary cancer risk locus or a randomly selected locus) as the dependent variable. Threshold of association was not a significant effect (*P*-value = 0.54). This result, that both associated and potentially associated breast cancer risk variants map more often to orthologs of rat mammary cancer risk loci than rat regions not associated with susceptibility, strongly supports that comparative genomics between humans and rats may be an effective integrative approach to determine which potential associations nominated by human association studies are true positives.

Human populations have been studied more extensively for breast cancer genetic risk than have rat populations; therefore, it is not surprising that human studies have yielded a considerable number of genome-wide significantly associated SNPs in alleles where it is not known if the rat genome contains a concordant allele. Interestingly, seven strongly associated human SNPs were in sequences orthologous to the randomly selected rat genome regions that are not known to associate with rat mammary cancer based on studies evaluating specific rat strains; thus, it is possible that a portion of the rat genome used in this study as rat random-genome control regions may actually associate with unidentified rat mammary cancer susceptibility loci. Thus, more rat genomic regions associated with mammary cancer risk may be identified with additional rat genetic studies. To date, only six inbred rat strains have been used to identify rat genomic regions associated with mammary cancer risk [15,16,20-23]. Therefore, it is highly likely that more mammary cancer susceptibility loci may be identified by incorporating additional rat strains. It is also possible that more extensive analysis of previously studied rat strains may yield additional susceptibility loci by using a higher density of genetic markers for example.

Twenty-one of the 24 known rat mammary cancer associated loci are orthologous to human loci containing SNPs that are either associated or potentially associated with breast cancer risk. Fourteen of the known rat mammary cancer associated loci are orthologous to human risk alleles marked by GWA study nominated SNPs reaching genome-wide significance. Human GWA study designs do not definitively determine causative genes or mechanisms. The laboratory rat is a versatile experimental organism to complement human studies of breast cancer. For example, inbred rat strains provide a model with reduced genetic variation that can be genetically manipulated and environmentally controlled. The overlap between human breast and rat mammary cancer susceptibility associated loci suggests rats can be used extensively to study genetically determined mechanisms and environment interactions that will translate directly to human breast cancer risk and prevention.

### Human GWA study nominated breast cancer risk SNPs map similarly to rat mammary cancer associated loci identified using 7,12-dimethylbenz[a]anthracene or beta-estradiol

**Figure 3:**
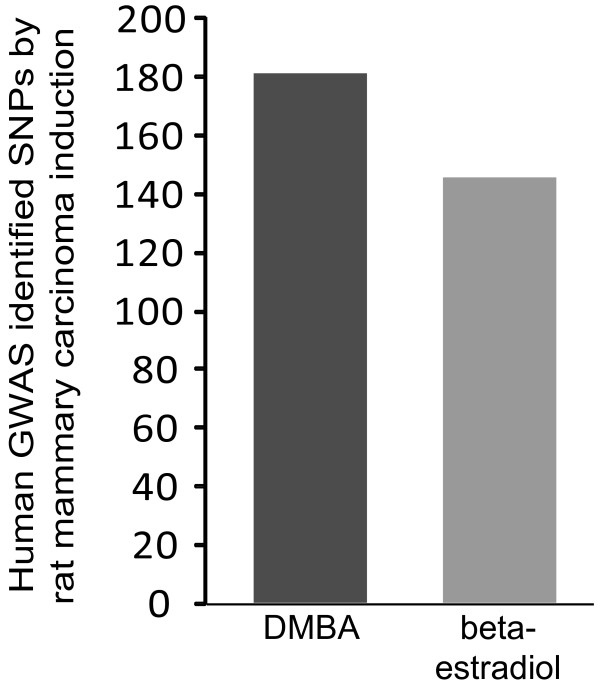
**Number of breast cancer risk GWA study nominated SNPs mapping to regions identified using DMBA or beta-estradiol.** Number of GWA study nominated SNPs mapping to rat mammary cancer loci separated by method of mammary carcinogenesis induction. Slightly more SNPs mapped to orthologs of rat loci that were identified using DMBA than beta-estradiol. DMBA, 7,12-dimethylbenz[a]anthracene; GWA, genome-wide association.

Several rat mammary cancer loci used in this study were identified using DMBA to induce mammary tumors. These are *Mcs1a-c, Mcs2-4, Mcs5a1, Mcs5a2, Mcs5b-c, Mcs6-Mcs8, Mcsm1, Mcstm1-2* and *Mcsta1.* The remaining rat mammary cancer loci considered were identified using beta-estradiol to induce mammary carcinogenesis. Estradiol-associated susceptibility loci are *Emca1-2* and *Emca4-8.* While DMBA is representative of environmental polycyclic aromatic hydrocarbons, this synthesized mammary carcinogen is not found in nature. Conversely, estradiol is an endogenous environmental exposure associated with breast cancer risk. Human GWA study nominated SNPs mapping to orthologs of rat mammary cancer loci identified using DMBA were compared to those identified using beta-estradiol. We considered SNPs from all GWA studies, irrespective of the population used. We noted that many DMBA and beta-estradiol identified rat mammary cancer loci overlap. In fact, seven of the fourteen DMBA associated rat mammary cancer loci overlap at least one beta-estradiol associated rat mammary cancer risk locus, and five of the seven beta-estradiol loci overlap rat mammary cancer loci identified using DMBA. To account for this overlap, human SNPs mapping to overlapping rat mammary cancer loci, one identified using DMBA and the other using beta-estradiol, were included once in the ‘DMBA’ group and once in the ‘beta-estradiol’ group. These results are shown in Figure 3. A relatively similar number of GWA study nominated SNPs mapped to orthologs of rat mammary cancer loci that were identified using DMBA (181 SNPs) and beta estradiol (146 SNPs). This suggests that different mammary carcinoma induction methods can effectively identify rat susceptibility loci relevant to human disease risk, and it also suggests that a plethora of carcinogenesis mechanisms may be genetically determined.

## Discussion

It has been suggested that the use of Bonferroni-based correction procedures to protect against multiple comparisons in GWA studies is too stringent and results in an abundance of false negative associations with little recourse to sort these from true-negative associations. Therefore, we considered associated and potentially associated human SNPs from breast cancer risk GWA studies to determine if SNPs that failed validation and SNPs that reached genome- wide significance map to respective regions of the rat genome known to associate with rat mammary cancer risk more often than to regions of the rat genome that are not known to associate with susceptibility. Results presented here indicate that the rat genome is useful to prioritize and rank human alleles potentially associated with risk. The rat genome is useful regardless of the human population studied. Significantly more SNPs from GWA studies of populations of European, Asian, and African-American descent map to human orthologous regions of rat mammary cancer loci than to human orthologous regions of randomly selected rat genomic regions not known to associate with mammary cancer susceptibility. This supports the general idea that there are SNPs associated with breast cancer risk that are missed due to conservative statistical methods used in GWA studies, and that the rat is useful to parse out important genetic variation in susceptibility to mammary carcinogenesis.

Interestingly, we were unable to map GWA study nominated SNPs to 3 of the 24 known rat mammary cancer loci. These were *Mcs1a, Mcs5a1* and *Mcs5c*. However, using a genome-targeted population-based genetic association study, a human SNP associated with breast cancer risk has been identified at human *MCS5A1*[9]. The risk-associated SNPs at *MCS5A1* are adjacent to a breast cancer risk-associated SNP at *MCS5A2*, which was identified in two independent human population based studies [9,43]. Taken together, there is a high correlation between genetics of breast cancer susceptibility in humans and mammary cancer susceptibility in rats. Interestingly, there are several human genomic regions that are human GWA study nominated hotspots (for example, 19q13, *FGFR2*) that are not known to have concordant rat orthologs. An explanation is that human breast and rat mammary cancer susceptibility are controlled by overlapping and non-overlapping genetic mechanisms. Another explanation is that there are rat genomic regions associated with mammary cancer risk yet to be discovered by using additional inbred strains, more extensive analysis of strains previously studied, and different methods of carcinogenesis induction.

## Conclusions

There is extensive genomic overlap between human breast and rat mammary cancer susceptibility. The rat genome may provide utility to identify true-positive associations regardless of the human population used for a GWA study. The laboratory rat will continue to be an important model organism for researching genetically determined mechanisms of mammary cancer susceptibility that may translate directly to human susceptibility. An appreciable number of GWA study nominated SNPs not meeting genome-wide significance levels have genomic overlap with rat mammary cancer susceptibility loci. This supports the general idea that Bonferroni-based multiple-comparison correction procedures are too stringent and complementary approaches that integrate rat genomics would be highly efficacious to prioritize breast cancer risk associated alleles.

## Abbreviations

ACI: August Copenhagen Irish; BN: Brown Norway; bp: base pair; BRCA 1 or 2: breast cancer 1 or 2; COP: Copenhagen; DMBA: 7,12-dimethylbenz[a]anthracene; EMCA: estrogen- induced mammary cancer; GWA study: genome-wide association study; Mcs: mammary carcinoma susceptibility; Mcsm: mammary carcinoma susceptibility modifier; Mcsta: mammary cancer susceptibility tumor aggressiveness; Mcstm: mammary cancer susceptibility tumor multiplicity; NMU: *N*-nitroso-*N*-methylurea; PALB 2: partner and localizer of BRCA2; QTL: quantitative trait locus; SNP: single nucleotide polymorphism; WF: Wistar-Furth; WKY: Wistar-Kyoto.

## Competing interests

The authors declare that they have no competing interests.

## Authors’ contributions

JS and DJS contributed to study conception and design, data analysis, and interpretation of results. JS and DJS drafted this manuscript and revised it. Both authors read and approved the final manuscript.

## Supplementary Material

Additional file 1: Table S1 List of GWAS nominated SNPs used in Analysis. Click here for file

Additional file 2: Table S2 Breast cancer risk associated polymorphisms from studies of European descent populations that map to rat mammary cancer loci and random rat regions. **Table S3**. Breast cancer risk associated polymorphisms from studies of non- European descent populations that map to rat mammary cancer loci and random rat regions. Click here for file
