# Supplementary material for: Significant overlap between human genome-wide association-study nominated breast cancer risk alleles and rat mammary cancer susceptibility loci
Source: Breast Cancer Res. 2014 Jan 27;16(1):R14. doi: 10.1186/bcr3607 (PMC4054882; doi:10.1186/bcr3607)
Supplement: Additional file 2: Table S2 — Breast cancer risk associated polymorphisms from studies of European descent populations that map to rat mammary cancer loci and random rat regions. Table S3. Breast cancer risk associated polymorphisms from studies of non- European descent populations that map to rat mammary cancer loci and random rat regions. [file bcr3607-S2.doc]

| **Supplemental Table 2. Breast cancer risk associated polymorphisms from studies of European descent populations that map to rat mammary cancer loci and random rat regions.** Underlined SNPs are found in both overlapping QTLs. Bold type SNPs reached genome-wide significance in the respective study. P-values are for last stage the respective SNP was tested. | | | | | |
| --- | --- | --- | --- | --- | --- |
| Locus | SNP | Position in human genome | OR (95%CI) | P-value | Reference |
| *Mcs1a* |  |  |  |  |  |
| *Mcs1b* | **rs889312** | *Chr5*: 56,031,884 | 1.13 (1.10-1.16) | 7E-20 | Easton et al (2007) |
|  | rs16886165 | *Chr5*: 56,023,083 | 1.23 (1.12–1.35) | 5E-07 | Thomas et al (2009) |
| *Mcs1c* | rs4704018 | *Chr5*: 84,263,712 |  | 0.044 | Ghoussaini et al (2012) |
| *Mcs2* | rs10507088 | *Chr12*: 97,879,744 |  | 0.00024 | Antoniou et al (2010) |
|  | rs7310517 | *Chr12*: 89,149,235 |  | 0.000565 | Antoniou et al (2010) |
|  | rs4807542 | *Chr19*: 1,104,078 | 1.10 (0.97-1.2) | 0.0001 | Mavvaddat et al (2009) |
|  | rs17740709 | *Chr12*: 83,423,340 | 0.91 (0.86-0.97) | 0.0024046 | Thomas et al (2009) |
|  | rs1154865 | *Chr12*: 73,989,837 |  |  | Murabito et al (2007) |
|  | rs4146372 | *Chr12*: 72,131,594 |  | 0.00007 | Murabito et al (2007) |
|  | rs2211914 | *Chr8*: 102,669,667 | 1.08 (1.02 to 1.14) | 4.96E-05 | Fletcher et al (2011) |
|  | rs4734571 | *Chr8*: 102,672,613 | 1.06 (1.01 to 1.12) | 1.30E-04 | Fletcher et al (2011) |
|  | rs2387620 | *Chr8*: 102,708,371 | 1.05 (1.00 to 1.11) | 5.90E-04 | Fletcher et al (2011) |
|  | rs6997395 | *Chr8*: 97,366,701 | 0.86 (0.77-0.96) | 0.0212 | Sehrawat et al (2011) |
|  | rs2546513 | *Chr12*: 69,108,448 | 0.89 (0.8-1.00) | 0.095 | Sehrawat et al (2011) |
|  | rs6991277 | *Chr8*: 97,400,151 | 1.04 (0.89-1.20) | 0.747 | Sehrawat et al (2011) |
|  | rs7818355 | *Chr8*: 97,358,968 | 1.01 (0.86-1.17) | 0.977 | Sehrawat et al (2011) |
|  | rs2053741 | *Chr8*: 102,140,698 | 0.87 (0.77-0.97) | 0.021 | Gaudet et al (2010) |
|  | **rs17356907** | *Chr12:* 96,027,759 |  | 1.8E-22 | Michailidou et al (2013) |
| *Mcs3* | rs4777792 | *Chr15*: 93,742,047 | 1.02 | 0.0033 | Ahmed et al (2009) |
|  | rs7110148 | *Chr11*: 20,653,783 |  | 2.96E-02 | Antoniou et al (2010) |
|  | rs11235127 | *Chr11*: 87,088,544 | 1.00 (0.90-1.11) | 0.41 | Easton et al (2007) |
|  | rs1546713 | *Chr15*: 99,471,866 | 0.94 (0.86-1.02) | 0.0039 | Mavvaddat et al (2009) |
|  | rs997669 | *Chr19*: 30,304,483 |  | 0.0034 | Mavvaddat et al (2009) |
|  | rs2229765 | *Chr15*: 99,478,225 |  | 0.0308 | Mavvaddat et al (2009) |
|  | rs3101649 | *Chr15*: 27,934,122 |  | 0.28 | Ghoussaini et al (2012) |
|  | rs7118677 | *Chr11*: 88,511,524 | 1.05 (0.94-1.18) | 0.483 | Sehrawat et al (2011) |
| *Mcs4* | rs956993 | *Chr11*: 131,354,471 | 1.02 | 0.00034 | Ahmed et al (2009) |
|  | rs7927483 | *Chr11*: 133,590,335 |  | 0.00018 | Antoniou et al (2010) |
|  | rs1783885 | *Chr11*: 124,693,144 |  | 0.00501 | Antoniou et al (2010) |
|  | rs550276 | *Chr11*: 125,446,070 |  | 3.89E-02 | Antoniou et al (2010) |
|  | rs2303659 | *Chr11*: 129,821,264 | 0.81 (0.72-0.91) | 0.00036 | Thomas et al (2009) |
|  | rs7116050 | *Chr11*: 130,637,654 | 0.9 (0.80-1.03) | 0.11 | Gaudet et al (2010) |
|  | rs7127732 | *Chr11*: 130,639,957 | 0.91 (0.80-1.03) | 0.13 | Gaudet et al (2010) |
|  | **rs7107217** | *Chr11*: 129,473,690 | 1.08 (1.05-1.11) | 4.6E-07 | Li et al (2011) |
|  | **rs11820646** | *Chr11:* 129,461,171 | 1.1E-09 |  | Michailidou et al (2013) |
| *Mcs5a1* |  |  |  |  |  |
| *Mcs5a2* | rs2182317 | *Chr9*: 37,641,851 |  | 0.0011 | Mavvaddat et al (2009) |
| *Mcs5b* | rs10512287 | *Chr9*: 104,458,968 |  |  | Murabito et al (2007) |
| *Mcs5c* |  |  |  |  |  |
| *Mcs6* | rs10507088 | *Chr12*: 97,879,744 |  | 0.00024 | Antoniou et al (2010) |
|  | rs7310517 | *Chr12*: 89,149,235 |  | 0.000565 | Antoniou et al (2010) |
|  | rs17740709 | *Chr12*: 83,423,340 | 0.91 (0.86-0.97) | 0.0024046 | Thomas et al (2009) |
|  | rs1154865 | *Chr12*: 73,989,837 |  |  | Murabito et al (2007) |
|  | rs4146372 | *Chr12*: 72,131,594 |  | 0.00007 | Murabito et al (2007) |
|  | **rs17356907** | *Chr12:* 96,027,759 |  | 1.8E-22 | Michailidou et al (2013) |
| *Mcs7* | **rs6504950** | *Chr17*: 53,056,471 | 0.95(0.92-0.97) | 1.4E-08 | Ahmed et al (2009) |
|  | rs7222197 | *Chr17*: 53,047,499 |  |  | Turnbull et al (2010) |
|  | rs1156287 | *Chr17*: 53,076,799 | 0.91 (0.85–0.97) | 0.0058 | Turnbull et al (2010) |
|  | **rs2075555** | *Chr17*: 48,274,291 |  | 8.3E-08 | Murabito et al (2007) |
|  | rs2532348 | *Chr17*: 44,340,253 |  | 0.000078 | Ghoussaini et al (2012) |
|  | rs199523 | *Chr17*: 44,848,517 |  | 0.0063 | Ghoussaini et al (2012) |
|  | rs1526123 | *Chr17*: 43,783,340 |  | 0.0038 | Ghoussaini et al (2012) |
|  | rs7206949 | *Chr17*: 44,247,017 |  | 0.024 | Ghoussaini et al (2012) |
|  | rs2668632 | *Chr17*: 44,319,366 |  | 0.00031 | Ghoussaini et al (2012) |
|  | rs4968451 | *Chr17*: 59,927,307 |  | 0.0048 | Ghoussaini et al (2012) |
|  | rs4522464 | *Chr17*: 60,199,130 |  | 0.014 | Ghoussaini et al (2012) |
|  | rs2233768 | *Chr17*: 67,144,576 |  | 0.93 | Ghoussaini et al (2012) |
|  | rs3803825 | *Chr17*: 51,901,084 | 1.24 (1.11-1.39) | 0.000118 | Gaudet et al (2010) |
|  | rs4794469 | *Chr17*: 51,909,049 | 1.22 (1.09-1.36) | 0.000251 | Gaudet et al (2010) |
|  | rs4483901 | *Chr17*:51,862,028 | 1.15 (1.06-1.25) | 0.000924 | Gaudet et al (2010) |
|  | **rs999737** | *Chr17*: 69,034,682 | 0.94 (0.88–0.99) | 1.74E-07 | Thomas et al (2009) |
| *Mcs8* | rs1881668 | *Chr4*: 70,725,456 | 1.05 (0.99-1.13) |  | Mavvaddat et al (2009) |
|  | rs3736599 | *Chr4*: 70,725,821 |  | 0.0053 | Mavvaddat et al (2009) |
|  | rs3775775 | *Chr4*: 70,718,282 |  | 0.0182 | Mavvaddat et al (2009) |
| *Mcsm1* | rs8021611 | *Chr14*: 27,613,621 | 0.93 | 0.015 | Ahmed et al (2009) |
|  | rs11850325 | *Chr14*: 27,626,887 | 0.90 | 0.035 | Ahmed et al (2009) |
|  | rs12882700 | *Chr14*: 66,372,429 |  | 0.00456 | Antoniou et al (2010) |
|  | rs17157070 | *Chr7*: 109,352,935 | 1.24 (0.90-1.70) | 0.1 | Easton et al (2007) |
|  | rs8009944 | *Chr14*: 69,039,588 | 0.88 (0.82–0.95) | 0.0004 | Turnbull et al (2010) |
|  | rs3784194 | *Chr14*: 32,923,626 |  | 0.3 | Ghoussaini et al (2012) |
|  | rs12433708 | *Chr14*: 63,937,832 | 0.99 (0.85-1.14) | 0.935 | Sehrawat et al (2011) |
|  | rs268840 | *Chr14*: 58,000,042 | 0.99 (0.89-1.11) | 0.967 | Sehrawat et al (2011) |
|  | **rs2236007** | *Chr14:* 37,132,729 |  | 1.7E-13 | Michailidou et al (2013) |
|  | **rs2588809** | *Chr14:* 68,660,428 |  | 1.4E-10 | Michailidou et al (2013) |
| *Mcstm1* | rs2182317 | *Chr9*: 37,641,851 |  | 0.0011 | Mavvaddat et al (2009 |
|  | rs10512287 | *Chr9*: 104,458,968 |  |  | Murabito et al (2007) |
|  | rs10993693 | *Chr9:* 93,557,698 | 1.12 (1.03-1.22) | 6.99E-03 | Gaudet et al (2010) |
|  | rs11138489 | *Chr9:* 82,713,431 | 0.87 (0.75-1.01) | 7.08E-02 | Sehrawat et al (2011) |
|  | rs12000794 | *Chr9:* 103,157,515 | 1.48 (1.25-1.75) | 5.40E-06 | Li et al (2010) |
|  | rs12350727 | *Chr9:* 94,264,616 |  | 0.039 | Ghoussaini et al (2012) |
|  | rs12553524 | *Chr9*: 93,569,646 | 0.90 (0.82-0.98) | 2.03E-02 | Gaudet et al (2010) |
|  | rs1319677 | *Chr9:* 93,565,999 | 0.89( 0.82-0.96) | 3.98E-03 | Gaudet et al (2010) |
|  | rs13299280 | *Chr9:* 82,690,543 | 0.92 (0.80-1.07) | 2.75E-01 | Sehrawat et al (2011) |
|  | rs1805787 | *Chr8:* 90,968,303 | 1.34 (1.10-1.65) |  | Mavvaddat et al (2009 |
|  | rs1932658 | *Chr9:* 86,815,750 |  | 0.79 | Ghoussaini et al (2012) |
|  | rs1952461 | *Chr9:* 79,222,584 |  | 0.53 | Ghoussaini et al (2012) |
|  | rs290274 | *Chr9:* 93,546,913 | 0.89 (0.82-0.97) | 6.69E-03 | Gaudet et al (2010) |
|  | rs3134615 | *Chr1:* 40,362,066 | 1.19 (1.05-1.35) | 0.0003 | Thomas et al (2009) |
|  | rs338230 | *Chr1:* 58,940,596 |  |  | Antoniou et al (2010) |
|  | rs3748648 | *Chr8:* 61,851,466 |  | 0.004798 | Li et al (2010) |
|  | rs4268201 | *Chr9:* 36,888,148 |  | 0.02 | Ghoussaini et al (2012) |
|  | rs4877564 | *Chr9:* 83,001,846 |  |  | Antoniou et al (2010) |
|  | rs4877566 | *Chr9:* 83,0.15,449 |  |  | Antoniou et al (2010) |
|  | rs6478296 | *Chr9:* 119,992,267 | 0.85 (0.77-0.95) | 2.74E-03 | Sehrawat et al (2011) |
|  | rs6479347 | *Chr9:* 94,268,566 |  |  | Murabito et al (2007) |
|  | rs667052 | *Chr9:* 110,882,390 | 1.08 (1.02-1.15) | 5.37E-05 | Fletcher et al (2011) |
|  | rs7030526 | *Chr9:* 111,022,525 | 0.89 (0.84-0.94) | 1.03E-05 | Fletcher et al (2011) |
|  | rs7036417 | *Chr9:* 93,570,505 | 1.17 (1.07-1.27) | 5.14E-04 | Gaudet et al (2010) |
|  | rs7047256 | *Chr9:* 83,027,012 |  | 7.42E-04 | Antoniou et al (2010) |
|  | rs786990 | *Chr9:* 115,416,257 | 0.90 (0.83-0.98) | 1.74E-02 | Gaudet et al (2010) |
|  | **rs865686** | *Chr9:* 110,888,478 | 0.89 (0.85-0.92) | 1.75E-10 | Fletcher et al (2011) |
|  | **rs10759243** | *Chr9:* 110,306,115 | 1.06 (1.03–1.08) | 1.2E-08 | Michailidou et al (2013) |
| *Mcstm2* | rs11150978 | *Chr18:* 74,441,415 | 0.91 (0.81-1.01) | 8.93E-02 | Gaudet et al (2010) |
|  | rs11660857 | *Chr18:* 74,437,679 | 0.91 (0.82-1.02) | 1.08E-01 | Gaudet et al (2010) |
|  | rs11877439 | *Chr18:* 48,291,329 |  | 0.89 | Antoniou et al (2010) |
|  | rs17735708 | *Chr18:* 48,284,174 |  | 1.24E-02 | Antoniou et al (2010) |
|  | rs1811455 | *Chr18:* 48,283,649 |  | 1.23E-02 | Antoniou et al (2010) |
|  | rs1978503 | *Chr18:* 53,664,282 |  |  | Murabito et al (2007) |
|  | rs380950 | *Chr18:* 10.362,359 |  |  | Antoniou et al (2010) |
|  | rs4536550 | *Chr18:* 70,948,491 |  | 0.54 | Ghoussaini et al (2012) |
|  | rs7243090 | *Chr18:* 12,273,032 | 0.89 (0.82-0.97) | 1.01E-02 | Gaudet et al (2010) |
|  | rs8095374 | *Chr18:* 43,793,488 | 0.99 (0.89-1.09) | 7.85E-01 | Sehrawat et al (2011) |
|  | rs8098165 | *Chr18:* 67,168,567 |  | 0.011 | Ghoussaini et al (2012) |
|  | rs920784 | *Chr18:* 48,318,120 |  | 2.32E-02 | Antoniou et al (2010) |
|  | rs9325024 | *Chr5:* 146,205,786 |  |  | Murabito et al (2007) |
|  | rs9967490 | *Chr18:* 72,229,212 |  | 2.35E-01 | Antoniou et al (2010) |
| *Mcsta1* | **rs6504950** | *Chr17*: 53,056,471 | 0.95(0.92-0.97) | 1.4E-08 | Ahmed et al (2009) |
|  | rs7222197 | *Chr17*: 53,047,499 |  |  | Turnbull et al (2010) |
|  | rs1156287 | *Chr17*: 53,076,799 | 0.91 (0.85–0.97) | 0.0058 | Turnbull et al (2010) |
|  | **rs2075555** | *Chr17*: 48,274,291 |  | 8.3E-08 | Murabito et al (2007) |
|  | rs2532348 | *Chr17*: 44,340,253 |  | 0.000078 | Ghoussaini et al (2012) |
|  | rs199523 | *Chr17*: 44,848,517 |  | 0.0063 | Ghoussaini et al (2012) |
|  | rs1526123 | *Chr17*: 43,783,340 |  | 0.0038 | Ghoussaini et al (2012) |
|  | rs7206949 | *Chr17*: 44,247,017 |  | 0.024 | Ghoussaini et al (2012) |
|  | rs2668632 | *Chr17*: 44,319,366 |  | 0.00031 | Ghoussaini et al (2012) |
|  | rs4968451 | *Chr17*: 59,927,307 |  | 0.0048 | Ghoussaini et al (2012) |
|  | rs4522464 | *Chr17*: 60,199,130 |  | 0.014 | Ghoussaini et al (2012) |
|  | rs2233768 | *Chr17*: 67,144,576 |  | 0.93 | Ghoussaini et al (2012) |
|  | rs3803825 | *Chr17*: 51,901,084 | 1.24 (1.11-1.39) | 0.000118 | Gaudet et al (2010) |
|  | rs4794469 | *Chr17*: 51,909,049 | 1.22 (1.09-1.36) | 0.000251 | Gaudet et al (2010) |
|  | rs4483901 | *Chr17*:51,862,028 | 1.15 (1.06-1.25) | 0.000924 | Gaudet et al (2010) |
|  | rs11077820 | *Chr17:* 74,454,668 | 0.97 (0.89-1.06) | 0.03 | Thomas et al (2009) |
|  | rs12051769 | *Chr17:* 11,977,952 | 1.18 (1.10-1.27) | 1.6E-05 | Turnbull et al (2010) |
|  | rs12658840 | *Chr5:* 160,318,988 |  |  | Easton et al (2007) |
|  | rs13160298 | *Chr5:* 157,796,317 |  | 0.15 | Ghoussaini et al (2012) |
|  | rs1801201 | *Chr17:* 37,879,585 |  | 0.0139 | Mavvaddat et al (2009) |
|  | rs1873062 | *Chr17:* 3,346,130 |  | 0.00095 | Ghoussaini et al (2012) |
|  | rs1990236 | *Chr17:* 11,865,462 |  | 0.00052 | Ghoussaini et al (2012) |
|  | rs2059273 | *Chr16:* 6,051,143 |  |  | Murabito et al (2007) |
|  | rs2080976 | *Chr5:* 166,991,039 | 1.12 (1.01-1.25) | 3.70E-02 | Sehrawat et al (2011) |
|  | rs231020 | *Chr17:* 15,162,389 |  | 0.22 | Ghoussaini et al (2012) |
|  | rs4074770 | *Chr17:* 36,022,817 |  |  | Li et al (2010) |
|  | rs6556756 | *Chr5:* 163,889,280 |  |  | Murabito et al (2007) |
|  | rs6994019 | *Chr8:* 89,268,543 |  | 2.71E-04 | Antoniou et al (2010) |
|  | rs734138 | *Chr16:* 2,999,218 |  | 4.29E-04 | Antoniou et al (2010) |
|  | rs757594 | *Chr17:* 12,000,632 | 0.99 (0.95-1.03) | 1.20E-05 | Turnbull et al (2010) |
|  | rs7711990 | *Chr5:* 180,374,898 |  |  | Murabito et al (2007) |
|  | rs8075722 | *Chr17:* 3,175,658 | 1.17 (0.98-1.38) | 8.12E-02 | Sehrawat et al (2011) |
|  | rs858524 | *Chr17:* 7,511,287 |  | 0.0329 | Mavvaddat et al (2009) |
|  | rs9314033 | *Chr5:* 163,389,206 |  |  | Murabito et al (2007) |
|  | rs9325024 | *Chr5:* 146,205,786 |  |  | Murabito et al (2007) |
|  | **rs1432679** | *Chr5:* 158,244,083 | 1.07 (1.05–1.09) | 2.0E-14 | Michailidou et al (2013) |
|  | **rs999737** | *Chr17*: 69,034,682 | 0.94 (0.88–0.99) | 1.74E-07 | Thomas et al (2009) |
| *Emca1* | **rs1011970** | *Chr9:* 22,062,134 | 1.09 (1.04–1.14) | 2.5E-08 | Turnbull et al (2010) |
|  | rs1857434 | *Chr9:* 19,980,632 | 1.09 (0.95-1.25) | 2.05E-01 | Sehrawat et al (2011) |
|  | rs3134615 | *Chr1:* 40,362,066 | 1.19 (1.05-1.35) | 0.0003 | Thomas et al (2009) |
|  | rs338230 | *Chr1:* 58,940,596 |  |  | Antoniou et al (2010) |
|  | rs3731239 | *Chr9:* 21,974,218 |  | 0.0122 | Mavvaddat et al (2009) |
| *Emca2* | rs11241138 | *Chr5:*111,142,040 |  | 0.0095 | Ghoussaini et al (2012) |
|  | rs380950 | *Chr18:* 10,362,359 |  |  | Antoniou et al (2010) |
|  | rs1978503 | *Chr18:* 53,664,282 |  |  | Murabito et al (2007) |
|  | rs7243090 | *Chr18:* 12,273,032 | 0.89 (0.82-0.97) | 1.01E-02 | Gaudet et al (2010) |
|  | rs9325024 | *Chr5:* 146,205,786 |  |  | Murabito et al (2007) |
| *Emca4* | rs2211914 | *Chr8*: 102,669,667 | 1.08 (1.02 to 1.14) | 4.96E-05 | Fletcher et al (2011) |
|  | rs4734571 | *Chr8*: 102,672,613 | 1.06 (1.01 to 1.12) | 1.30E-04 | Fletcher et al (2011) |
|  | rs2387620 | *Chr8*: 102,708,371 | 1.05 (1.00 to 1.11) | 5.90E-04 | Fletcher et al (2011) |
|  | rs6997395 | *Chr8*: 97,366,701 | 0.86 (0.77-0.96) | 0.0212 | Sehrawat et al (2011) |
|  | rs6991277 | *Chr8*: 97,400,151 | 1.04 (0.89-1.20) | 0.747 | Sehrawat et al (2011) |
|  | rs7818355 | *Chr8*: 97,358,968 | 1.01 (0.86-1.17) | 0.977 | Sehrawat et al (2011) |
|  | rs2053741 | *Chr8*: 102,140,698 | 0.87 (0.77-0.97) | 0.021 | Gaudet et al (2010) |
|  | rs1026411 | *Chr8:*128,026,410 | 1.00 (0.92-1.08) | 4.20E-01 | Gaudet et al (2010) |
|  | **rs13281615** | *Chr8:*128,355,618 |  |  | Easton et al (2007) |
|  | **rs1562430** | *Chr8:* 128,382,852 | 0.84 (0.78 to 0.90) | 3.15E-11 | Fletcher et al (2011) |
|  | rs6469633 | *Chr8:* 117,136,227 |  |  | Easton et al (2007) |
|  | rs672888 | *Chr8:* 128,345,463 | 1.18 (1.05–1.33) | 4.98E−03 | Li et al (2011) |
| *Emca5* | rs1876206 | *Chr15*: 48,900,586 |  |  | Murabito et al (2007) |
|  | rs10501093 | *Chr11*: 27,990,119 |  |  | Murabito et al (2007) |
|  | rs7116850 | *Chr11*: 27,645,530 |  | 0.73 | Ghoussaini et al (2012) |
|  | rs6493076 | *Chr15*: 43,318,140 | 0.89 (0.75-1.04) | 0.234 | Sehrawat et al (2011) |
|  | rs10469689 | *Chr2:*164,260,063 |  | 0.0083 | Ghoussaini et al (2012) |
|  | rs1119133 | *Chr20:*11,303,203 |  | 5.24E-01 | Antoniou et al (2010) |
|  | rs165557 | *Chr20:* 12,898,764 |  | 7.40E-02 | Antoniou et al (2010) |
|  | rs311499 | *Chr20:* 62,217,589 | 0.72 (0.61-0.85) | 6.64E-05 | Gaudet et al (2010) |
|  | rs3935234 | *Chr20:* 22,447,954 | 0.94 (0.83-1.06) | 2.93E-01 | Sehrawat et al (2011) |
|  | rs406193 | *Chr20:* 31,399,643 |  | 0.0026 | Mavvaddat et al (2009) |
|  | rs4810108 | *Chr20:* 56,449,176 |  | 4.48E-02 | Antoniou et al (2010) |
|  | rs6027564 | *Chr20:* 58,956,161 |  | 0.11 | Ghoussaini et al (2012) |
|  | rs6033101 | *Chr20:* 11,297,353 |  | 7.55E-02 | Antoniou et al (2010) |
|  | rs6059504 | *Chr20:* 32,457,158 |  | 5.89E-03 | Antoniou et al (2010) |
|  | rs6095541 | *Chr20:* 48,110,188 | 1.32 (1.11-1.56) |  | Mavvaddat et al (2009) |
|  | rs6095543 | *Chr20:* 48,113,300 |  | 0.0016 | Mavvaddat et al (2009) |
|  | rs6101183 | *Chr20:* 59,591,960 |  |  | Murabito et al (2007) |
|  | rs6108719 | *Chr20:* 10,834,078 |  | 3.70E-02 | Antoniou et al (2010) |
|  | rs6134722 | *Chr20:*12,908,451 |  | 7.10E-03 | Antoniou et al (2010) |
|  | rs693649 | *Chr20:* 48,188,079 | 0.69 (0.53-0.88) |  | Mavvaddat et al (2009) |
|  | rs732417 | *Chr20:* 54,967,065 |  | 0.0182 | Mavvaddat et al (2009) |
|  | rs7936636 | *Chr11:* 44,440,970 | 1.10 (0.86-1.44) | 0.65 | Thomas et al (2009) |
|  | rs8122608 | *Chr20:* 11,317,483 |  | 1.16E-02 | Antoniou et al (2010) |
|  | rs896617 | *Chr11:* 40,329,199 | 0.85 (0.77-0.93) | 5.22E-04 | Gaudet et al (2010) |
|  | rs9630178 | *Chr11:* 39,703,309 | 1.25 (1.07-1.47) | 5.47E-03 | Sehrawat et al (2011) |
|  | **rs2016394** | *Chr2:* 172,972,971 | (0.93–0.97) | 1.2E-08 | Michailidou et al (2013) |
|  | **rs1550623** | *Chr2:* 174,212,894 | 0.94 (0.92–0.97) | 3.0E-08 | Michailidou et al (2013) |
| *Emca6* | rs9307064 | *Chr4*: 90,146,649 |  |  | Murabito et al (2007) |
|  | rs10486490 | *Chr7:*26,073,549 |  |  | Murabito et al (2007) |
|  | rs10487920 | *Chr7:* 146,458,079 |  |  | Murabito et al (2007) |
|  | rs13240597 | *Chr7:* 23,284,961 |  | 1.05E-02 | Antoniou et al (2010) |
|  | rs2433294 | *Chr7:* 131,199,903 | 1.14 (1.03-1.27) | 9.95E-03 | Gaudet et al (2010) |
|  | rs6442221 | *Chr3:* 1,171,860 |  |  | Antoniou et al (2010) |
|  | rs6971207 | *Chr7:* 139,520,063 | 1.85 (1.23-2.70) |  | Mavvaddat et al (2009) |
|  | rs7577855 | *Chr2:* 81,346,430 |  | 3.80E-03 | Antoniou et al (2010) |
|  | rs7646994 | *Chr3:* 1,170,794 |  | 4.27E-02 | Antoniou et al (2010) |
|  | rs996424 | *Chr3:* 1,165,633 |  | 4.46E-03 | Antoniou et al (2010) |
|  | **rs6762644** | *Chr3:* 4,742,276 | 1.07 (1.04–1.09) | 2.2E-12 | Michailidou et al (2013) |
|  | **rs720475** | *Chr7:* 144,074,929 | 0.94 (0.92–0.96) | 7.0E-11 | Michailidou et al (2013) |
| *Emca7* | rs11677670 | *Chr2:* 25,510,276 | 0.68 (0.52-0.87) |  | Mavvaddat et al (2009) |
|  | rs8021611 | *Chr14*: 27,613,621 | 0.93 | 0.015 | Ahmed et al (2009) |
|  | rs11850325 | *Chr14*: 27,626,887 | 0.90 | 0.035 | Ahmed et al (2009) |
|  | rs12882700 | *Chr14*: 66,372,429 |  | 0.00456 | Antoniou et al (2010) |
|  | rs8009944 | *Chr14*: 69,039,588 | 0.88 (0.82–0.95) | 0.0004 | Turnbull et al (2010) |
|  | rs3784194 | *Chr14*: 32,923,626 |  | 0.3 | Ghoussaini et al (2012) |
|  | rs12433708 | *Chr14*: 63,937,832 | 0.99 (0.85-1.14) | 0.935 | Sehrawat et al (2011) |
|  | rs268840 | *Chr14*: 58,000,042 | 0.99 (0.89-1.11) | 0.967 | Sehrawat et al (2011) |
|  | **rs2236007** | *Chr14:* 37,132,729 |  | 1.7E-13 | Michailidou et al (2013) |
|  | **rs2588809** | *Chr14:* 68,660,428 |  | 1.4E-10 | Michailidou et al (2013) |
|  | rs17030257 | *Chr2:* 43,232,117 |  | 0.00063 | Ahmed et al (2009) |
|  | rs343155 | *Chr2:* 20,592,798 |  |  | Stacey et al (2007) |
|  | rs3738863 | *Chr2:* 30,457,561 |  | 0.00059 | Ghoussaini et al (2012) |
|  | rs4666451 | *Chr2:* 19,286,943 | 0.97(0.94–1.00) | 6.00E-05 | Easton et al (2007) |
| *Emca8* | **rs1011970** | *Chr9:* 22,062,134 | 1.09 (1.04–1.14) | 2.5E-08 | Turnbull et al (2010) |
|  | rs10512287 | *Chr9*: 104,458,968 |  |  | Murabito et al (2007) |
|  | rs10993693 | *Chr9:* 93,557,698 | 1.12 (1.03-1.22) | 6.99E-03 | Gaudet et al (2010) |
|  | rs11138489 | *Chr9:* 82,713,431 | 0.87 (0.75-1.01) | 7.08E-02 | Sehrawat et al (2011) |
|  | rs12000794 | *Chr9:* 103,157,515 | 1.48 (1.25-1.75) | 5.40E-06 | Li et al (2010) |
|  | rs12350727 | *Chr9:* 94,264,616 |  | 0.039 | Ghoussaini et al (2012) |
|  | rs12553524 | *Chr9*:93,569,646 | 0.90 (0.82-0.98) | 2.03E-02 | Gaudet et al (2010) |
|  | rs1319677 | *Chr9:* 93,565,999 | 0.89( 0.82-0.96) | 3.98E-03 | Gaudet et al (2010) |
|  | rs13299280 | *Chr9:* 82,690,543 | 0.92 (0.80-1.07) | 2.75E-01 | Sehrawat et al (2011) |
|  | rs1932658 | *Chr9:* 86,815,750 |  | 0.79 | Ghoussaini et al (2012) |
|  | rs1952461 | *Chr9:* 79,222,584 |  | 0.53 | Ghoussaini et al (2012) |
|  | rs290274 | *Chr9:* 93,546,913 | 0.89 (0.82-0.97) | 6.69E-03 | Gaudet et al (2010) |
|  | rs4268201 | *Chr9:* 36,888,148 |  | 0.02 | Ghoussaini et al (2012) |
|  | rs4877564 | *Chr9:* 83,001,846 |  |  | Antoniou et al (2010) |
|  | rs4877566 | *Chr9:* 83,0.15,449 |  |  | Antoniou et al (2010) |
|  | rs6478296 | *Chr9:* 119,992,267 | 0.85 (0.77-0.95) | 2.74E-03 | Sehrawat et al (2011) |
|  | rs6479347 | *Chr9:* 94,268,566 |  |  | Murabito et al (2007) |
|  | rs667052 | *Chr9:* 110,882,390 | 1.08 (1.02-1.15) | 5.37E-05 | Fletcher et al (2011) |
|  | rs7030526 | *Chr9:* 111,022,525 | 0.89 (0.84-0.94) | 1.03E-05 | Fletcher et al (2011) |
|  | rs7036417 | *Chr9:* 93,570,505 | 1.17 (1.07-1.27) | 5.14E-04 | Gaudet et al (2010) |
|  | rs7047256 | *Chr9:* 83,027,012 |  | 7.42E-04 | Antoniou et al (2010) |
|  | **rs865686** | *Chr9:* 110,888,478 | 0.89 (0.85-0.92) | 1.75E-10 | Fletcher et al (2011) |
|  | **rs10759243** | *Chr9:* 110,306,115 | 1.06 (1.03–1.08) | 1.2E-08 | Michailidou et al (2013) |
|  | rs2182317 | *Chr9*: 37,641,851 |  | 0.0011 | Mavvaddat et al (2009 |
|  | rs786990 | *Chr9:* 115,416,257 | 0.90 (0.83-0.98) | 1.74E-02 | Gaudet et al (2010) |
|  | rs1857434 | *Chr9:* 19,980,632 | 1.09 (0.95-1.25) | 2.05E-01 | Sehrawat et al (2011) |
|  | rs3731239 | *Chr9:* 21,974,218 |  | 0.0122 | Mavvaddat et al (2009) |
|  | rs3134615 | *Chr1:* 40,362,066 | 1.19 (1.05-1.35) | 0.0003 | Thomas et al (2009) |
|  | rs338230 | *Chr1:* 58,940,596 |  |  | Antoniou et al (2010) |
| Random Rat Region 1 | rs1257022 | *Chr2*: 97,614,936 | 1.03 | 0.000074 | Ahmed et al (2009) |
|  | rs9454109 | *Chr6*: 67,947,988 |  | 0.0178 | Antoniou et al (2010) |
|  | rs12622050 | *Chr2*: 101,579,454 | 0.90 (0.85-0.96) | 0.0031813 | Thomas et al (2009) |
| Random Rat Region 2 | rs6561682 | *Chr13*: 53,266,297 | 1.07 (0.96-1.20) | 0.311 | Sehrawat et al (2011) |
| Random Rat Region 3 | rs2391406 | *Chr13*: 107,788,872 | 0.97 (0.93-1.01) | 0.1120642 | Thomas et al (2009) |
|  | rs9586525 | *Chr13*: 105,124,679 |  | 0.016 | Ghoussaini et al (2012) |
|  | rs2617076 | *Chr8:* 4,445,174 |  | 0.31 | Ghoussaini et al (2012) |
| Random Rat Region 4 | rs1378506 | *Chr5*: 109,391,230 | 1.03 | 0.00014 | Ahmed et al (2009) |
|  | rs4854071 | *Chr2*: 240,885,050 |  | 0.000308 | Antoniou et al (2010) |
|  | rs1138444 | *Chr2*: 240,946,766 |  | 0.000325 | Antoniou et al (2010) |
|  | rs4149556 | *Chr2:* 240,915,307 |  | 0.000575 | Antoniou et al (2010) |
|  | rs2289407 | *Chr2*: 240,954,110 |  | 0.000574 | Antoniou et al (2010) |
|  | rs12606686 | *Chr18*: 9,042,622 |  | 0.95 | Ghoussaini et al (2012) |
| Random Rat Region 5 | rs16861794 | *Chr1*: 183,920,191 |  | 0.0629 | Antoniou et al (2010) |
|  | rs3820260 | *Chr1*: 181,638,511 | 1.21 | 0.000087 | Stacey et al (2007) |
| Random Rat Region 6 | rs4132417 | *Chr3*: 117,426,258 | 1.00 (0.97–1.03) | 0.016 | Ahmed et al (2009) |
|  | rs1401083 | *Chr3*: 116,373,807 |  | 0.0874 | Antoniou et al (2010) |
|  | rs9879234 | *Chr3*: 115,100,216 | 0.9 (0.82-0.98) | 0.0126 | Gaudet et al (2010) |
|  | rs13071097 | *Chr3*: 102,938,744 | 0.92 (0.84-1.00) | 0.0448 | Gaudet et al (2010) |
| Random Rat Region 7 | rs10508468 | *Chr10*: 13,918,753 | 0.97(0.91-1.03) | 0.02 | Easton et al (2007) |
|  | rs2184380 | *Chr10*: 8,714,074 | 1.2 | 0.0000052 | Stacey et al (2007) |
|  | **rs2380205** | *Chr10*: 5,886,734 | 0.86 (0.81-0.92) | 0.25 | Turnbull et al (2010) |
|  | rs7074055 | *Chr10*: 12,625,436 |  | 0.15 | Ghoussaini et al (2012) |
|  | rs7099921 | *Chr10*: 13,126,019 | 1.15 (1.01-1.31) | 0.0896 | Sehrawat et al (2011) |
|  | rs11257153 | *Chr10*: 11,569,824 | 0.89 (0.77-1.04) | 0.223 | Sehrawat et al (2011) |
|  | rs11254759 | *Chr10*: 6,857,307 | 0.8 (0.71-0.91) | 0.000429 | Gaudet et al (2010) |
|  | rs7895644 | *Chr10*: 6,858,920 | 0.81 (0.71-0.91) | 0.00051 | Gaudet et al (2010) |
|  | rs11254771 | *Chr10*: 6,862,055 | 0.81 (0.72-0.92) | 0.00103 | Gaudet et al (2010) |
|  | rs10906720 | *Chr10*: 14,675,475 | 0.83 (0.73-0.94) | 0.00367 | Gaudet et al (2010) |
|  | **rs7072776** | *Chr10:* 22,032,942 |  | 4.3E-14 | Michailidou et al (2013) |
|  | **rs11814448** | *Chr10:* 22,315,843 |  | 9.3E-16 | Michailidou et al (2013) |
| Random Rat Region 8 | rs13400898 | *Chr2*: 154,875,613 |  | 0.5 | Ghoussaini et al (2012) |
|  | rs4455975 | *Chr9*: 129,383,199 |  | 0.00056 | Ghoussaini et al (2012) |
| Random Rat Region 9 | rs3852789 | *Chr16*: 72,228,506 | 0.96 (0.87-1.06) | 0.00005 | Easton et al (2007) |
|  | rs1294255 | *Chr1*: 233,501,358 |  |  | Murabito et al (2007) |
|  | rs1274466 | *Chr10*: 34,470,204 | 0.94 (0.88-1.01) | 0.0096863 | Thomas et al (2009) |
|  | **rs1981867** | *Chr16*: 80,923,769 | 1.22 (1.09-1.36) | 0.000432 | Sehrawat et al (2011) |
|  | **rs13329835** | *Chr16:* 80,650,805 |  | 2.1E-16 | Michailidou et al (2013) |
| Random Rat Region 10 | rs7307700 | *Chr12*: 125,588,197 | 1.04 (0.98-1.09) | 0.002 | Easton et al (2007) |
|  | rs10850145 | *Chr12*: 113,944,129 | 1.12 (1.04-1.20) | 0.0004547 | Thomas et al (2009) |
|  | rs10263639 | *Chr7*: 67,059,267 |  |  | Murabito et al (2007) |
|  | **rs1292011** | *Chr12*: 115,836,522 |  | 4.3E-19 | Ghoussaini et al (2012) |
|  | rs7955262 | *Chr12*: 116,206,962 |  | 0.0042 | Ghoussaini et al (2012) |
|  | rs3108049 | *Chr7*: 67,742,720 | 0.83 (0.75-0.92) | 0.000649 | Gaudet et al (2010) |
|  | rs6489171 | *Chr12*: 127,882,841 | 0.84 (0.77–0.90) | 6.88E-06 | Li et al (2010) |
| Random Rat Region 11 | rs12333016 | *Chr6*: 107,099,191 |  | 0.45 | Ghoussaini et al (2012) |
|  | rs9401003 | *Chr6*: 117,718,303 |  | 0.00065 | Ghoussaini et al (2012) |
| Random Rat Region 12 |  |  |  |  |  |
| Random Rat Region 13 | rs9491859 | *Chr6:*149,064,363 | 1.04 (0.98-1.11) | 0.006 | Thomas et al (2009) |
|  | rs9497624 | *Chr6:* |  | 7.22E-02 | Antoniou et al (2010) |
| Random Rat Region 14 | rs11249433 | *Chr1:*  121,280,612 |  |  | Li et al (2010) |
|  | **rs11552449** | *Chr1:* 114,448,388 |  | 1.8E-08 | Michailidou et al (2013) |

| **Supplemental Table 3. Breast cancer risk associated polymorphisms nominated from GWA studies of non-European descent populations that map to rat mammary cancer loci or random rat regions.** Underlined SNPs are found in both overlapping rat QTLs. Bold type SNPs reached genome-wide significance in the respective study. P-values are for the last stage the respective SNP was tested. | | | | | | |
| --- | --- | --- | --- | --- | --- | --- |
| Locus | SNP | Position in human genome | OR (95%CI) | P-value | Reference | Population |
| *Mcs1a* |  |  |  |  |  |  |
| *Mcs1b* |  |  |  |  |  |  |
| *Mcs1c* | rs376689 | *Chr5*: 82,299,073 | 1.16(1.07- 1.26) | 0.0036 | Cai et al (2011) | Asian descent |
| *Mcs2* | rs1732534 | *Chr12*: 67,634,919 | 0.95(0.87- 1.03) | 0.014 | Cai et al (2011) | Asian descent |
|  | **rs17356907** | *Chr12:* 96,027,759 | 0.93 (0.90-0.97) | 1.99E-04 | Zheng et al (2013) | Asian descent |
| *Mcs3* | rs977835 | *Chr15*: 98,700,918 |  | 0.000017 | Chen et al (2012) | African- American descent |
|  | rs10899287 | *Chr11*: 76,514,335 | 1.08(0.98- 1.19) | 0.00019 | Cai et al (2011) | Asian descent |
|  | rs11073746 | *Chr15*: 88,361,491 | 1.17(1.07- 1.29) | 0.0054 | Cai et al (2011) | Asian descent |
|  | rs4453257 | *Chr11*: 79,814,276 |  |  | Long et al (2010) | Asian descent |
| *Mcs4* | rs2085421 | *Chr11*: 129473690 | 1.08 (1.05-1.11) | 4.6E-07 | Chen et al (2012) | African- American descent |
|  | rs4564353 | *Chr11*: 128,425,884 | 1.17(1.04- 1.32) | 0.066 | Cai et al (2011) | Asian descent |
|  | rs11820646 | *Chr11:* 129,461,171 | 1.05 (1.01-1.08) | 0.004 | Zheng et al (2013) | Asian descent |
|  | rs7107217 | *Chr11:* 129,473,690 |  |  | Long et al (2012) | Asian descent |
|  | rs8026506 | *Chr15:* 67,608,155 |  | 4.0E-05 | Chen et al (2012) | African- American descent |
| *Mcs5a1* |  |  |  |  |  |  |
| *Mcs5a2* |  |  |  |  |  |  |
| *Mcs5b* |  |  |  |  |  |  |
| *Mcs5c* |  |  |  |  |  |  |
| *Mcs6* | **rs17356907** | *Chr12:* 96,027,759 | 0.93 (0.90-0.97) | 1.99E-04 | Zheng et al (2013) | Asian descent |
| *Mcs7* | rs8068469 | *Chr17*: 67,837,662 |  | 6.1E-05 | Chen et al (2012) | African- American descent |
|  | rs11077488 | *Chr17:* 68,290,082 |  |  | Kim et al (2012) | Asian descent |
| Mcs8 |  |  |  |  |  |  |
| Mcsm1 | rs1950510 | *Chr14*: 50,644,200 | 1.11(0.93-1.33) | 0.57 | Zheng et al (2009) | Asian descent |
|  | rs990864 | *Chr14*: 50,699,549 | 1.02(0.87-1.20) | 0.93 | Zheng et al (2009) | Asian descent |
|  | rs216531 | *Chr14*: 60,642,970 | 1.01(0.85-1.20) | 0.7 | Zheng et al (2009) | Asian descent |
|  | rs10483813 | *Chr14*: 69,031,284 |  |  | Kim et al (2012) | Asian descent |
|  | rs762063 | *Chr14*: 69,031,284 |  | 0.00079 | Chen et al (2012) | African- American descent |
|  | rs3784179 | *Chr14*: 32,852,903 | 0.99(0.88- 1.12) | 0.71 | Cai et al (2011) | Asian descent |
|  | rs12436827 | *Chr14*: 60,281,387 | 0.99(0.91- 1.08) | 0.051 | Cai et al (2011) | Asian descent |
|  | rs1123204 | *Chr14*: 37,219,269 |  |  | Long et al (2010) | Asian descent |
|  | **rs2236007** | *Chr14:* 37,132,769 | 0.92 (0.89-0.95) | 5.61E-06 | Zheng et al (2013) | Asian descent |
|  | rs2588809 | *Chr14:* 68,660,428 | 0.94 (0.86-1.04) | 0.225 | Zheng et al (2013) | Asian descent |
| *Mcstm1* | rs10761309 | *Chr9:* 96,753.722 | 0.94(0.89-1.00) | 0.05 | Long et al (2010) | Asian descent |
|  | rs10781090 | Chr9: 74,839,689 | 1.06 (0.85-1.32) | 0.793 | Zheng et al (2009) | Asian descent |
|  | rs10889221 | *Chr1:* 61,872,541 |  |  | Long et al (2012) | Asian descent |
|  | rs10983070 | *Chr9:* 118,919,075 | 1.13 (0.76-1.69) | 0.97 | Zheng et al (2009) | Asian descent |
|  | rs11583352 | *Chr1:* 30,945,486 |  | 2.4E-05 | Chen et al (2012) | African- American descent |
|  | rs1235390 | *Chr8:* 91,269,272 | 1.06 (0.60-1.88) | 0.39 | Zheng et al (2009) | Asian descent |
|  | rs12403009 | *Chr1:* 29,047,110 | 0.87( 0.74- 1.03) | 1.6E-02 | Cai et al (2011) | Asian descent |
|  | rs12727993 | *Chr1:* 61,841,528 | 0.83( 0.73- 0.95) | 7.4E-04 | Cai et al (2011) | Asian descent |
|  | rs3176626 | *Chr9:* 100,460,643 |  |  | Long et al (2012) | Asian descent |
|  | rs422481 | *Chr1:* 39,529,134 | 0.95(0.89-1.01) | 0.1 | Long et al (2010) | Asian descent |
|  | rs4738723 | *Chr8:* 59,776,637 |  | 5.9E-06 | Chen et al (2012) | African- American descent |
|  | rs6560285 | *Chr9:* 75,150,031 | 0.92(0.87-0.97) | 3.4E-03 | Long et al (2010) | Asian descent |
|  | rs7520470 | *Chr1:* 59,891,821 | 0.84( 0.74- 0.95) | 6.1E-03 | Cai et al (2011) | Asian descent |
|  | rs7825751 | *Chr8:* 59,410,755 |  | 1.0E-05 | Chen et al (2012) | African- American descent |
|  | rs9436636 | *Chr1:* 61,812,773 | 0.96 (0.88-1.06) | 4.5E-01 | Kim et al (2012) | Asian descent |
|  | **rs10759243** | *Chr9:* 110,306,115 | 0.95 (0.91-0.99) | 0.008 | Zheng et al (2013) | Asian descent |
| *Mcstm2* | rs11875595 | *Chr18:* 42,308,972 | 1.14( 0.99- 1.31) | 2.9E-03 | Cai et al (2011) | Asian descent |
|  | rs16975392 | *Chr18:* 10,812,573 | 0.93(0.87-0.98) | 9.1E-03 | Long et al (2010) | Asian descent |
|  | rs4147513 | *Chr18:* 45,946,275 | 0.99(0.93-1.06) | 0.82 | Long et al (2010) | Asian descent |
|  | rs8097442 | *Chr18:* 45,885,036 |  | 1.4E-05 | Chen et al (2012) | African- American descent |
| *Mcsta1* | rs8068469 | *Chr17*: 67,837,662 |  | 6.1E-05 | Chen et al (2012) | African- American descent |
|  | rs10479046 | *Chr5:* 132,917,556 | 1.18 (1.07-1.30) | 7.2E-04 | Long et al (2010) | Asian descent |
|  | rs11077488 | *Chr17:* 68,290,082 | 0.89 (0.76-1.03) | 0.16 | Kim et al (2012) | Asian descent |
|  | rs12162135 | *Chr17:* 30,805,795 |  | 4.2E-05 | Chen et al (2012) | African- American descent |
|  | rs12451931 | *Chr17:* 13,139,119 |  | 2.0E-04 | Chen et al (2012) | African- American descent |
|  | rs13172733 | *Chr5:* 142,371,680 |  | 2.4E-06 | Chen et al (2012) | African- American descent |
|  | rs153170 | *Chr5:* 142,277,251 |  | 4.1E-06 | Chen et al (2012) | African- American descent |
|  | rs17096232 | *Chr5:* 154,224,949 |  | 1.7E-05 | Chen et al (2012) | African- American descent |
|  | rs17116409 | *Chr5:* 154,121,691 |  | 1.2E-05 | Chen et al (2012) | African- American descent |
|  | rs315791 | *Chr5:* 169,735,920 | 1.10( 0.97- 1.24) | 6.9E-02 | Cai et al (2011) | Asian descent |
|  | rs3822397 | *Chr5:* 142,393,562 |  | 7.7E-05 | Chen et al (2012) | African- American descent |
|  | rs4976412 | *Chr5:* 136,449,984 |  |  | Long et al (2012) | Asian descent |
|  | rs617384 | *Chr5:* 141,417,287 | 1.22( 1.09- 1.37) | 1.2E-03 | Cai et al (2011) | Asian descent |
|  | rs6880837 | *Chr5:* 135,396,669 |  | 1.9E-06 | Chen et al (2012) | African- American descent |
|  | rs7203563 | *Chr16:* 6,078,181 | 1.32 (1.11-1.57) | 0.57 | Gold et al (2008) | Ashkenazi Jewish descent |
|  | rs7443571 | *Chr5:* 169,520,419 |  |  | Long et al (2012) | Asian descent |
|  | rs7716019 | *Chr5:* 154,148,862 |  | 3.8E-04 | Chen et al (2012) | African- American descent |
|  | rs8078837 | *Chr17:* 30,763,537 |  | 3.8E-05 | Chen et al (2012) | African- American descent |
|  | rs876302 | *Chr5:* 169,734,070 | 1.19( 1.01- 1.41) | 7.0E-03 | Cai et al (2011) | Asian descent |
|  | **rs1432679** | *Chr5:* 158,244,083 | 0.92 (0.89-0.96) | 2.79E-06 | Zheng et al (2013) | Asian descent |
| *Emca1* | rs11583352 | *Chr1:* 30,945,486 |  | 2.4E-05 | Chen et al (2012) | African- American descent |
|  | rs12403009 | *Chr1:* 29,047,110 | 0.87( 0.74- 1.03) | 1.6E-02 | Cai et al (2011) | Asian descent |
|  | rs12727993 | *Chr1:* 61,841,528 | 0.83( 0.73- 0.95) | 7.4E-04 | Cai et al (2011) | Asian descent |
|  | rs422481 | *Chr1:* 39,529,134 | 0.95(0.89-1.01) | 0.1 | Long et al (2010) | Asian descent |
|  | rs7520470 | *Chr1:* 59,891,821 | 0.84( 0.74- 0.95) | 6.1E-03 | Cai et al (2011) | Asian descent |
|  | rs9436636 | *Chr1:* 61,812,773 | 0.96 (0.88-1.06) | 4.5E-01 | Kim et al (2012) | Asian descent |
|  | rs10889221 | *Chr1:* 61,872,541 |  |  | Long et al (2012) | Asian descent |
| *Emca2* | rs13172733 | *Chr5:* 142,371,680 |  | 2.4E-06 | Chen et al (2012) | African- American descent |
|  | rs153170 | *Chr5:* 142,277,251 |  | 4.1E-06 | Chen et al (2012) | African- American descent |
|  | rs16975392 | *Chr18:* 10,812,573 | 0.93(0.87-0.98) | 9.1E-03 | Long et al (2010) | Asian descent |
|  | rs3822397 | *Chr5:* 142,393,562 |  | 7.7E-05 | Chen et al (2012) | African- American descent |
|  | rs617384 | *Chr5:* 141,417,287 | 1.22( 1.09- 1.37) | 1.2E-03 | Cai et al (2011) | Asian descent |
| *Emca4* | **rs13281615** | *Chr8:* 128,355,618 |  |  | Kim et al (2012) | Asian descent |
| *Emca5* | rs1367814 | *Chr15*: 46,798,142 | 1.23( 1.12- 1.35) | 0.000022 | Cai et al (2011) | Asian descent |
|  | rs11033111 | *Chr11:* 35,422,796 |  |  | Long et al (2012) | Asian descent |
|  | rs2073124 | *Chr20:* 49,870,811 | 1.11( 0.96- 1.28) | 4.4E-02 | Cai et al (2011) | Asian descent |
|  | rs297709 | *Chr20:* 4,388,115 |  |  | Long et al (2012) | Asian descent |
|  | rs4383110 | *Chr15:* 34,036,470 | 0.95(0.89-1.02) | 0.19 | Long et al (2010) | Asian descent |
|  | rs4516385 | *Chr2:* 181,062,674 | 1.03(0.97-1.09) | 0.4 | Long et al (2010) | Asian descent |
|  | rs6100755 | *Chr20:* 58,725,838 | 0.95(0.90-1.00) | 0.07 | Long et al (2010) | Asian descent |
|  | rs628583 | *Chr20:* 4,386,485 |  |  | Long et al (2012) | Asian descent |
|  | rs7580896 | *Chr2:* 181,083,696 | 1.01 (0.90-1.13) | 8.6E-01 | Kim et al (2012) | Asian descent |
|  | rs2016394 | *Chr2:* 172,972,971 | 0.99 (0.94-1.05) | 0.790 | Zheng et al (2013) | Asian descent |
|  | rs1550623 | *Chr2:* 174,212,894 | 1.21 (1.00-1.47) | 0.054 | Zheng et al (2013) | Asian descent |
| *Emca6* | rs2597540 | *Chr4*: 121,746,232 | 1.02 (0.93-1.12) | 0.65 | Kim et al (2012) | Asian descent |
|  | rs6834599 | *Chr4*: 122,078,180 |  | 2.4E-05 | Chen et al (2012) | African- American descent |
|  | rs13116936 | *Chr4*: 122,126,808 |  | 1.1E-05 | Chen et al (2012) | African- American descent |
|  | rs2869950 | *Chr4*: 89,742,764 | 1.10(1.01-1.19) | 2.7E-05 | Long et al (2010) | Asian descent |
|  | rs10251934 | *Chr7:* 144,702,577 | 0.99 (0.89-1.10) | 8.1E-01 | Kim et al (2012) | Asian descent |
|  | **rs10510333** | *Chr3:* 6,416,779 | 1.15 | 1.5E-05 | Chen et al (2012) | African- American descent |
|  | rs1806667 | *Chr7:* 133,726,092 | 1.08(1.02-1.14) | 0.01 | Long et al (2010) | Asian descent |
|  | rs2471214 | *Chr7:* 122,260,557 |  |  | Long et al (2012) | Asian descent |
|  | rs7627541 | *Chr3:* 126,980,437 |  | 3.8E-04 | Chen et al (2012) | African- American descent |
|  | rs985343 | *Chr2:* 76,911,782 | 1.18( 1.05- 1.33) | 8.0E-03 | Cai et al (2011) | Asian descent |
|  | rs6762644 | *Chr3:* 4,742,276 | 1.03 (0.98-1.09) | 0.286 | Zheng et al (2013) | Asian descent |
|  | rs720475 | *Chr7:* 144,074,929 | 0.98 (0.89-1.07) | 0.619 | Zheng et al (2013) | Asian descent |
| *Emca7* | rs13425772 | *Chr2:* 31,454,609 |  | 3.1E-05 | Chen et al (2012) | African- American descent |
|  | rs1950510 | *Chr14*: 50,644,200 | 1.11(0.93-1.33) | 0.57 | Zheng et al (2009) | Asian descent |
|  | rs990864 | *Chr14*: 50,699,549 | 1.02(0.87-1.20) | 0.93 | Zheng et al (2009) | Asian descent |
|  | rs216531 | *Chr14*: 60,642,970 | 1.01(0.85-1.20) | 0.7 | Zheng et al (2009) | Asian descent |
|  | rs10483813 | *Chr14*: 69,031,284 |  |  | Kim et al (2012) | Asian descent |
|  | rs762063 | *Chr14*: 69,031,284 |  | 0.00079 | Chen et al (2012) | African- American descent |
|  | rs3784179 | *Chr14*: 32,852,903 | 0.99(0.88- 1.12) | 0.71 | Cai et al (2011) | Asian descent |
|  | rs12436827 | *Chr14*: 60,281,387 | 0.99(0.91- 1.08) | 0.051 | Cai et al (2011) | Asian descent |
|  | rs1123204 | *Chr14*: 37,219,269 |  |  | Long et al (2010) | Asian descent |
|  | **rs2236007** | *Chr14:* 37,132,769 | 0.92 (0.89-0.95) | 5.61E-06 | Zheng et al (2013) | Asian descent |
|  | rs2588809 | *Chr14:* 68,660,428 | 0.94 (0.86-1.04) | 0.225 | Zheng et al (2013) | Asian descent |
| *Emca8* | rs10511591 | *Chr9:* 14,141,071 |  |  | Long et al (2012) | Asian descent |
|  | rs10761309 | *Chr9:* 96,753.722 | 0.94(0.89-1.00) | 0.05 | Long et al (2010) | Asian descent |
|  | rs10781090 | Chr9: 74,839,689 | 1.06 (0.85-1.32) | 0.793 | Zheng et al (2009) | Asian descent |
|  | rs10810557 | *Chr9:* 16,336,250 | 1.06 (0.60-1.88) | 0.39 | Zheng et al (2009) | Asian descent |
|  | rs10889221 | *Chr1:* 61,872,541 |  |  | Long et al (2012) | Asian descent |
|  | rs10983070 | *Chr9:* 118,919,075 | 1.13 (0.76-1.69) | 0.97 | Zheng et al (2009) | Asian descent |
|  | rs6560285 | *Chr9:* 75,150,031 | 0.92(0.87-0.97) | 3.4E-03 | Long et al (2010) | Asian descent |
|  | **rs10759243** | *Chr9:* 110,306,115 | 0.95 (0.91-0.99) | 0.008 | Zheng et al (2013) | Asian descent |
|  | rs3176626 | *Chr9:* 100,460,643 |  |  | Long et al (2012) | Asian descent |
|  | rs422481 | *Chr1:* 39,529,134 | 0.95(0.89-1.01) | 0.1 | Long et al (2010) | Asian descent |
|  | rs7520470 | *Chr1:* 59,891,821 | 0.84( 0.74- 0.95) | 6.1E-03 | Cai et al (2011) | Asian descent |
|  | rs9436636 | *Chr1:* 61,812,773 | 0.96 (0.88-1.06) | 4.5E-01 | Kim et al (2012) | Asian descent |
|  | rs12727993 | *Chr1:* 61,841,528 | 0.83( 0.73- 0.95) | 7.4E-04 | Cai et al (2011) | Asian descent |
| Random Rat Region 1 |  |  |  |  |  |  |
| Random Rat Region 2 | rs7333229 | *Chr13*: 60,211,498 |  |  | Long et al (2010) | Asian descent |
| Random Rat Region 3 | rs6474389 | *Chr8*: 42,187,712 |  | 1.4E-04 | Chen et al (2012) | African- American descent |
|  | rs2740873 | *Chr8*: 3,921,821 | 0.90(0.82-0.98) | 0.38 | Long et al (2010) | Asian descent |
| Random Rat Region 4 | rs11674289 | Chr2: 241,121,411 | 0.93(0.79- 1.08) | 0.43 | Zheng et al (2009) | Asian descent |
|  | rs12515340 | *Chr5*: 106,269,629 | 0.89(0.75-1.06) | 0.26 | Zheng et al (2009) | Asian descent |
|  | rs1549092 | *Chr18*: 1,535,957 |  | 2.6E-05 | Chen et al (2012) | African- American descent |
|  | rs1420864 | *Chr5*: 103,417,386 | 1.02(0.93-1.11) | 0.08 | long et al (2010) | Asian descent |
| Random Rat Region 5 | rs3861950 | *Chr1*: 173,156,292 |  | 1.1E-06 | Chen et al (2012) | African- American descent |
|  | rs1234317 | *Chr1*: 173,187,775 |  | 4.8E-05 | Chen et al (2012) | African- American descent |
| Random Rat Region 6 |  |  |  |  |  |  |
| Random Rat Region 7 | rs4750190 | *Chr10*: 6,299,773 |  | 0.00014 | Chen et al (2012) | African- American descent |
|  | rs10159718 | *Chr10*: 1,139,183 | 0.94(0.89-1.00) | 0.03 | Long et al (2010) | Asian descent |
|  | rs1218370 | *Chr10*: 14,327,174 |  |  | Long et al (2010) | Asian descent |
|  | rs10827927 | *Chr10*: 20,308,656 |  |  | Long et al (2010) | Asian descent |
|  | rs7072776 | *Chr10:* 22,032,942 | 0.96 (0.87-1.06) | 0.424 | Zheng et al (2013) | Asian descent |
|  | rs1181448 | *Chr10:* 22,315,843 | 1.08 (0.90-1.30) | 0.389 | Zheng et al (2013) | Asian descent |
| Random Rat Region 8 | rs3829849 | *Chr9*: 129,390,800 |  |  | Long et al (2010) | Asian descent |
| Random Rat Region 9 | rs1381499 | *Chr16*: 82,835,886 |  | 2.9E-05 | Chen et al (2012) | African- American descent |
|  | rs11640522 | *Chr16*: 82,800,368 |  | 5.7E-05 | Chen et al (2012) | African- American descent |
|  | rs9932176 | *Chr16*: 74,907,440 |  | 4.0E-05 | Chen et al (2012) | African- American descent |
|  | rs13329835 | *Chr16:* 80,650,805 | 1.02 (0.96-1.10) | 0.483 | Zheng et al (2013) | Asian descent |
| Random Rat Region 10 | rs11609565 | *Chr12*: 132,093,024 | 0.90 (0.77-1.06) | 0.334 | Zheng et al (2009) | Asian descent |
| Random Rat Region 11 | rs9320374 | *Chr6*: 111,983,818 | 1.04 (0.94-1.14) | 0.46 | Kim et al (2012) | Asian descent |
| Random Rat Region 12 |  |  |  |  |  |  |
| Random Rat region 13 | **rs9485372** | *Chr6*:  149,608,873 | 0.89 (0.85–0.94) | 3.8E-12 | Long et al (2012) | Asian descent |
|  | rs9498283 | *Chr6:*  149,605,181 | 0.92 (0.79-1.07) | 9.6E-03 | Kim et al (2012) | Asian descent |
| Random rat region 14 | rs11249433 | *Chr1:*  121,280,612 | 1.16 (1.05–1.28) | 0.005 | Zheng et al (2013) | Asian descent |
|  | rs11552449 | *Chr1:* 114,448,388 | 1.03 (0.99-1.06) | 0.131 | Zheng et al (2013) | Asian descent |
